# Transition metal transporting P‐type ATPases: terminal metal‐binding domains serve as sensors for autoinhibitory tails

**DOI:** 10.1111/febs.17330

**Published:** 2024-11-28

**Authors:** Qiaoxia Hu, Oleg Sitsel, Viktoria Bågenholm, Christina Grønberg, Pin Lyu, Anna Sigrid Pii Svane, Kasper Røjkjær Andersen, Nick Stub Laursen, Gabriele Meloni, Poul Nissen, Dennis W. Juhl, Jakob Toudahl Nielsen, Niels Chr. Nielsen, Pontus Gourdon

**Affiliations:** ^1^ Department of Biomedical Sciences University of Copenhagen Denmark; ^2^ Department of Molecular Biology and Genetics Aarhus University Denmark; ^3^ Interdisciplinary Nanoscience Center (iNANO) and Department of Chemistry Aarhus University Denmark; ^4^ Department of Chemistry and Biochemistry The University of Texas at Dallas Richardson TX USA; ^5^ Department of Experimental Medical Science Lund University Sweden; ^6^ Present address: Marine Structural Biology Unit Okinawa Institute of Science and Technology Graduate University Onna Japan

**Keywords:** autoinhibition, copper transport, metal‐binding domains, P‐type ATPases, regulation

## Abstract

Copper is an essential micronutrient and yet is highly toxic to cells at elevated concentrations. P_1B_‐ATPase proteins are critical for this regulation, providing active extrusion across cellular membranes. One unique molecular adaptation of P_1B_‐ATPases compared to other P‐type ATPases is the presence of metal‐binding domains (MBDs) at the cytosolic termini, which however are poorly characterized with an elusive mechanistic role. Here we present the MBD architecture in metal‐free and metal‐bound forms of the archetype Cu^+^‐specific P_1B_‐ATPase LpCopA, determined using NMR. The MBD is composed of a flexible tail and a structured core with a metal ion binding site defined by three sulfur atoms, one of which is pertinent to the so‐called CXXC motif. Furthermore, we demonstrate that the MBD rather than being involved in ion delivery likely serves a regulatory role, which is dependent on the classical P‐type ATPase E1‐E2 transport mechanism. Specifically, the flexible tail appears responsible for autoinhibition while the metal‐binding core is used for copper sensing. This model is validated by a conformation‐sensitive and MBD‐targeting nanobody that can structurally and functionally replace the flexible tail. We propose that autoinhibition of Cu^+^‐ATPases occurs at low copper conditions via MBD‐mediated interference with the soluble domains of the ATPase core and that metal transport is enabled when copper levels rise, through metal‐induced dissociation of the MBD. This allows P_1B_‐ATPase ‘vacuum cleaners’ to tune their own activity, balancing the levels of critical micronutrients in the cells.

## Graphical abstract

Copper is essential, yet toxic. High levels of copper are removed from the cells by proteins called P_1B_‐ATPases that have poorly understood metal‐binding domains (MBDs). Here, we present MBD structures from the P_1B_‐ATPase LpCopA, where a metal‐binding core senses copper conditions, enabling a flexible tail to block copper export at low concentrations and to allow export at higher copper levels. Thus, these ‘vacuum cleaners’ tune their own activity and balance cellular copper levels.

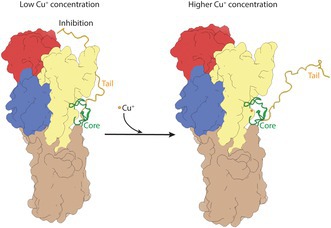

**Abbreviations**
A‐domain: actuator domain
AfCopA: P_1B‐1_‐ATPase from *Archaeoglobus fulgidus*
BME: β‐mercaptoethanol
BSA: bovine serum albumin
ELISA: enzyme‐linked immunosorbent assay
HSQC: heteronuclear single quantum correlation
ICP‐MS: inductively coupled plasma‐mass spectrometry
IRA: inferential restraint assignments
LpCopA: P_1B‐1_‐ATPase from *Legionella pneumophila*
LpMBD: N‐terminal metal‐binding domain region of LpCopA
LpMBD^core^
: structured N‐terminal region of LpCopA, residues 35–68
LpMBD^tail^
: N‐terminal tail of LpCopA, residues 1–34
MBDs: metal‐binding domains
MWCO: molecular weight cutoff
Nbs: nanobodies
NCS: non‐crystallographic symmetry
N‐domain: nucleotide‐binding domain
NOESY: nuclear overhauser effect spectroscopy
PAE: predicted aligned error
P‐domain: phosphorylation domain
PMBCs: peripheral blood mononuclear cells
PMCAs: plasma membrane Ca^2+^‐ATPases
RD: residue difference
RMSD: root mean square deviation
RND: resistance‐nodulation‐cell division
SERCA: sarcoendoplasmic reticulum Ca^2+^‐ATPase
SilB N‐MBD: N‐terminus of the MBD of the adaptor protein component of tripartite SilCBA metal exporters
TCEP: tris(2‐carboxyethyl)phosphine
TEV: tobacco etch virus
TMB: 3,3′,5,5′‐tetramethyl‐benzidine
WT: wild type

## Introduction

Copper is an essential transition metal in living cells, serving as a co‐factor for enzymes involved in many important biological processes [1]. Conversely, copper overload causes free radical‐induced damage on the cellular level and disease on the organismal level [2, 3]. Carefully balancing copper concentrations is therefore crucial, and copper transporting P‐type ATPases (P_1B‐1_‐ATPases, also known as CopA proteins) are central in this process, providing efflux of copper from cells and cellular compartments [4]. These membrane‐bound enzymes are found in all kingdoms of life and are important for a wide variety of physiological processes such as growth and development. Accordingly, malfunction of the two human P_1B‐1_‐ATPases, ATP7A or ATP7B, causes the severe Menkes and Wilson disorders [5, 6, 7], respectively.

**Fig. 1:**
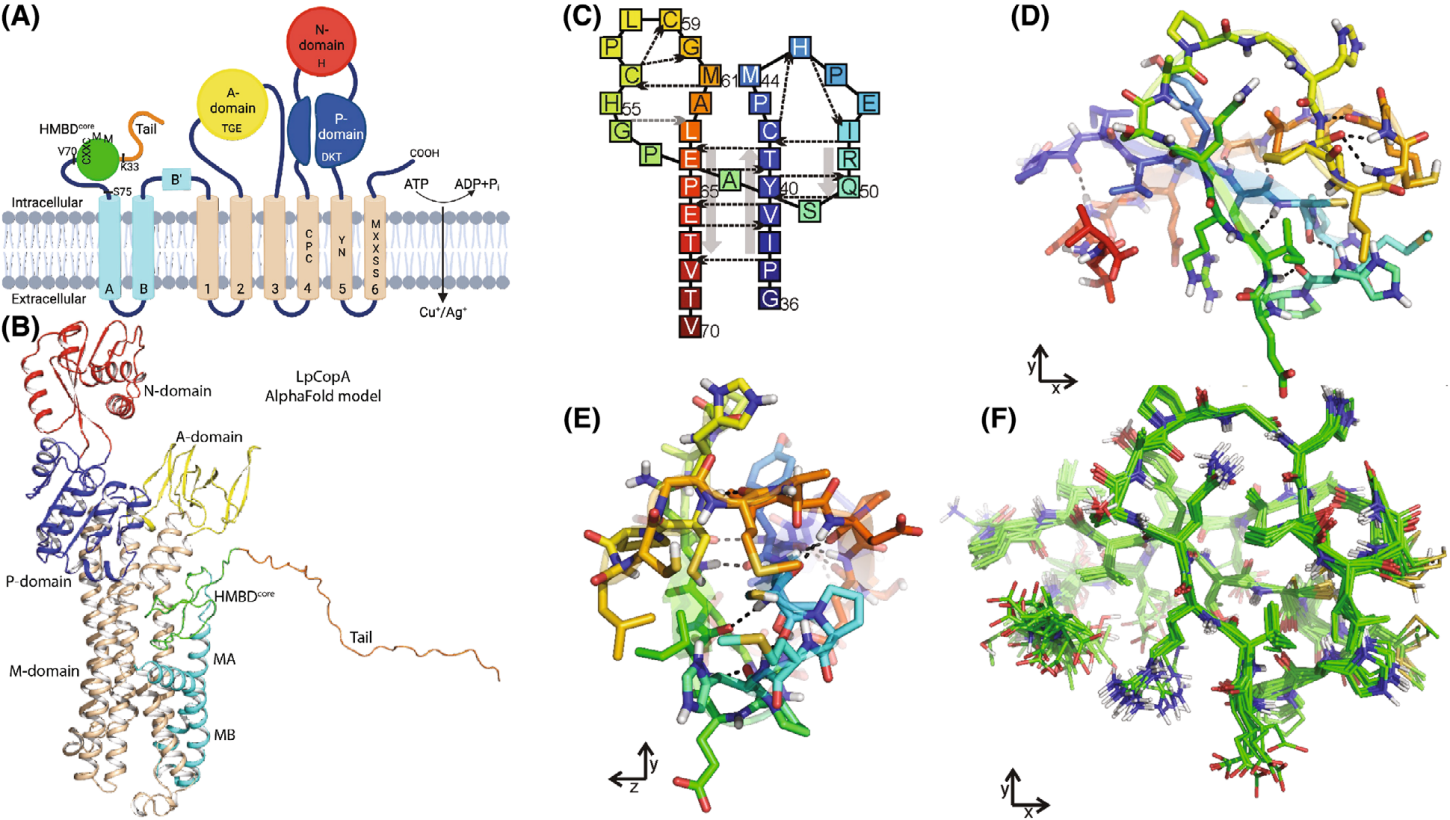
Overview of P_1B_‐ATPases including structure of the metal‐binding domain (MBD). (A) Topology of P_1B_‐ATPases with three cytosolic domains, A (actuator, with the TGE loop critical for dephosphorylation, shown in yellow), P (phosphorylation, with the DKT motif for phosphorylation, blue) and N (nucleotide‐binding, red), and eight transmembrane helices, MA and MB as well as and M1–M6 shown in cyan and wheat, respectively. The MBD core and preceding tail are shown in green and orange. The Cu^+^/Ag^+^ transport direction of P_1B‐1_‐ATPAses such as LpCopA is indicated. Constructs of LpCopA included LpMBD^tail^ (residues 1–33), LpMBD^core^ (34–70), T0 (75–736), T1 (69–736) and T2 (34–736); the approximate starts and ends of these forms are indicated. (B) Alphafold model of the copper‐transporting P_1B_‐ATPase LpCopA, colored as in panel A (model confidence seen in Fig. S1). (C–F) NMR structure of the metal‐free form of the LpMBD^core^. (C) Topology diagram of the structure with the position in sequence highlighted with a color ramp highlighting β‐sheets in gray arrows and hydrogen bonds using black dashed arrows from C=O to H_N_. (D, E) Cartoon representations of the lowest energy structure of the LpMBD^core^ showing residues as sticks and H‐bonds with black dashes (F). An ensemble of the 10 best structural models of the LpMBD^core^ showing C, O, H, and N atoms in green, red, white, and blue lines, respectively. The panels with structures were generated using Pymol.

P‐type ATPases encompass a broad superfamily of ATP‐dependent active transporters of ions, lipids, polyamines and helices, and based on sequence alignments, they are divided into five major classes (P_1_‐P_5_‐ATPases) that also distinguish cargo specificity [4, 8, 9]. P‐type ATPases exploit a transport mechanism with four principal states, named E1, E1P, E2P, and E2, which includes ATP‐mediated phosphorylation of the enzyme during the E1 to E1P step transition, and auto‐dephosphorylation during transition from E2P to E2 [9]. These modifications cause P‐type ATPases to undergo conformational changes that ferry cargo against concentration gradients across lipid membranes. A general core architecture is maintained among all P‐type ATPases and includes three cytosolic domains: the A‐ (actuator, with a conserved TGE motif responsible for dephosphorylation), N‐ (nucleotide‐binding, with an ATP‐binding pocket), and P‐domain (phosphorylation, with the conserved DKTG motif including a catalytic aspartate residue that becomes phosphorylated during the reaction cycle) (Fig. 1A). In addition, a transmembrane M‐domain is present with six core helices, M1–M6, found in all P‐type ATPases, and which contain motifs that determine cargo specificity and partake in transport [10]. Many P‐type ATPase classes also have unique features such as regulatory domains and/or extra transmembrane helices. Considering the large cargo range, with significantly different chemical properties and availability in cells, as well as the resulting topological differences between classes, adaptations of the reaction cycle have taken place [10, 11]. As an example, P_1B_‐ATPases display several subclass‐specific features, including two transmembrane helices MA and MB, which likely are involved in ion uptake, and which are located to the N‐terminus of the conserved M‐domain core helices [12, 13]. Another distinctive property of P_1B_‐ATPases are the MBDs, which are typically present in the N‐termini [12]. Finally, many P_1B‐1_‐ATPases act in concert with metallochaperones such as CopZ (called Atox1 in humans) that sequester and deliver Cu^+^ to the ATPases [14, 15, 16], reflecting that the cellular copper levels are much lower and more tightly controlled compared to the typically non‐sequestered cargo of other P‐type ATPases.

Flexible and poorly conserved extensions such as MBDs at the N‐ and/or C‐terminal ends that flank the ATPase core represent an emerging hotspot for class‐specific modifications of P‐type ATPases [17]. Sometimes these are also subject to alternative splicing, leaving several different possible terminal segments, as observed for plasma membrane Ca^2+^‐ATPases (PMCAs) [18]. Accumulating evidence suggests that these stretches serve a regulatory function, as shown for all five main classes of P‐type ATPases such as PMCA calcium ATPases [19] (N‐terminal regulatory domain in plants, C‐terminal in mammals), proton pumps (C‐terminal) [20, 21, 22, 23], lipid flippases (C‐terminal) [24], and polyamine transporters (N‐terminal) [25]. The understanding of these parts of P‐type ATPases has been limited by the fact that these termini until recently have not been visible in structures of full‐length P‐type ATPases, suggesting that the termini are flexible at least in some conformations. Lately however, high‐resolution cryo‐electron microscopy structures of P_4_‐ and P_5_‐ATPases have pinpointed the structure of such autoinhibitory domains, which intercalates between the three soluble domains and thereby blocks the transport cycle [24]. Nevertheless, due to low sequence similarity of the N‐termini, it remains unclear if P‐type ATPases even within the same subclass share similar principles of regulation using their flanking extensions.

As the number of MBDs in P_1B_‐ATPases varies from one or two in prokaryotes to six in human, it is possible that additional MBDs confer more fine‐tuned regulation of the function, provided the role of these domains is indeed regulatory. Most investigated MBDs have a ferredoxin‐like βαββαβ fold with exposed CXXC motifs for ion binding, sharing a similar architecture to CopZ metallochaperones [14, 15, 16]. However, alternative MBD folds also exist, including the unusual cupredoxin‐like MBD found in a P_1B‐1_‐ATPase from *Streptococcus pneumoniae* [26]. The first structural glimpse of the location of the MBDs relative to the P‐type ATPase core was recently obtained through cryo‐EM studies of frog and human ATP7B [27, 28], something that had previously been indicated in biochemical studies [29]. These indicated a dynamic role of the most core‐proximal MBD, located at the A‐domain in the E2P state (for the frog protein) and at the MB helix in the E1 conformation (human ATP7B), while the preceding MBD was positioned to the N‐ and P‐domain interface in the E2P configuration (frog). However, a complete view of the role of the MBDs is lacking, not only because the structural location and dynamics during turnover and detailed functional role for several MBDs remain elusive, but also because only a few snapshots of the reaction cycle have been covered. Moreover, it is poorly understood if the MBDs of the eukaryotic and prokaryotic members serve similar purposes. The MBDs were not visible in the other available structures of P_1B_‐ATPases, which are limited to the E2P and dephosphorylation transition E2.P_i_ states of the copper‐transporting LpCopA from *Legionella pneumophila* [30, 31], and the zinc‐transporting SsZntA from *Shigella sonnei* [32] as well as the E1 conformation of the P_1B‐1_‐ATPase AfCopA from *Archaeoglobus fulgidus* [13].

Previous studies have proposed the MBDs to be either directly involved in ion uptake or in activity regulation [11, 33, 34, 35, 36, 37, 38]. The activity of AfCopA is unaffected by mutation or deletion of the two MBDs [34, 35], and CopZ from the same organism delivers Cu^+^ directly to the transmembrane domain of the transporter, a function that cannot be performed by isolated MBDs *in vitro* [33], while it has been observed for the yeast Ccc2 *in vivo* [37]. A recent study on human ATP7B suggests that the first three MBDs serve an autoinhibitory role, with release of the autoinhibition being controlled by acceptance of Cu^+^ from either the chaperone Atox1 or from isolated MBDs [36, 39]. Perplexingly, when isolated all six human MBDs can replace Atox1 by delivering Cu^+^ to wild‐type ATP7B for ion transport, whereas only isolated MBD6 (the core‐proximal domain) can directly deliver Cu^+^ to the M‐domain of a version of ATP7B that lacks all six MBDs [36]. The MBD in the copper‐transporting P‐type ATPase Ccc2 from yeast has been proposed to play a similar role, accepting Cu^+^ from the Atx1 chaperone, and to then transferring the ion to the entry site of the translocation pathway in the M‐domain [37]. For the Cd^2+^, Co^2+^, and Zn^2+^ transporting P_1B‐4_‐ATPase CzcP from *C. metallidurans* (a species lacking identified metallochaperones), the two classical ferredoxin‐like MBDs in the N‐terminus were proposed to have separate functions, one serving as a chaperone for ion delivery to the M‐domain, and the other as a metal sensor [38]. Functional studies on the P_1B‐2_‐ATPase SsZntA have shown that the activity is decreased by 50% when the MBD is removed or the CXXC motif is mutated [32], in line with an ion delivery or autoinhibitory function of the MBD. For LpCopA, it has been reported the protein loses almost all activity upon removal of the MBD [30]. Collectively, conflicting structural and biochemical data are available on the MBDs, and hence, there is still a discussion on their role for the P_1B_‐ATPases.

Here, we determine the structure of the MBD of LpCopA and discern structural features linked to metal binding. These efforts are complemented through identification of a nanobody that can partially replace the MBD, and additional experiments to further dissect the roles of the domain. Collectively, these data shed critical new light on the function of the MBD.

## Results and discussion

### Fold prediction of the type of MBD present in LpCopA

We have previously reported that the N‐terminal MBD of LpCopA may be critical for ion transport, perhaps providing cytoplasmic copper to the ATPase core for transfer across the membrane, but the molecular basis for its effects remains elusive [30]. The structure of the N‐terminal MBD present in LpCopA (LpMBD, residues M1‐V74) has not been determined, and the sequence shows no homology with available structures of MBDs except for a CXXC motif, which typically is associated with transition metal binding. To further dissect the roles of the MBDs in P_1B_‐ATPases, we turned to Alphafold analysis [40, 41]. The Alphafold model indicates the MBD of LpCopA consists of a low model confidence N‐terminal tail (LpMDB^tail^), indicating disorder or flexibility, and a structured core domain, exposing the cysteines of the CXXC motif (Fig. 1B). According to the model, LpMBD^tail^ roughly covers residues M1‐M34, and flexibility of this stretch is supported by secondary structure analyses using prediction software such as IUPRED [42] (Fig. S2). Conversely, the core portion of the MBD establishes a β‐sheet bundle and stretches approximately residues E35‐V68, thus linking almost directly to the determined LpCopA ATPase core structure, which starts with V74 of transmembrane helix MA [30]. As such, the structural part of LpMBD would represent the shortest identified MBD.

### NMR structure determination

To experimentally derive a high‐resolution structure of LpMBD and to assess structural changes upon metal binding, we turned to NMR, which is well‐suited for structural and dynamic characterization of relatively small soluble protein domains that contain abundant flexible regions. We overproduced and purified a ^13^C, ^15^N‐labeled version of LpMBD encompassing residues M1‐R84, the latter representing the beginning of the ATPase core at the membrane spanning part of helix MA, using established procedures, based on recombinant protein production in *E. coli* and purification by affinity‐ and size‐exclusion chromatography (Fig. 1A & Materials and Methods). Indeed, analysis of the assigned NMR chemical shifts revealed the presence of both structured as well as unstructured portions of the domain (Figs S3 and S4). All resonances for the structured form were assigned sequence specifically, and further analysis confirmed that residues M1‐G36 and V74‐R84 were disordered, whereas residues P37‐V73 establish a folded shape (Figs S4 and S1), which we denote LpMBD^core^. The structure of the latter was calculated using distance constraints derived from 3D NOESY correlation spectra.

### The structure of the LpMBD core

The elucidated structure, LpMBD^core^, consists of a β‐sheet with three anti‐parallel strands connected by two loops (Fig. 1C–F). The first β‐strand (I38‐T41) is present in the middle of the sheet, and the following loop contains both an open turn (C42‐H45) and a type I β‐turn (H45‐I48). This is followed by a short β‐strand of residues R49 and Q50 and an extended conformation of residues S51‐P53 initiating the second loop. This loop contains two stem forming residues at either end, G54‐H55 and A62‐L63, and a rubredoxin knuckle‐like turn for C56‐M61. The latter harbors a CPXCGX motif, which coordinates metal ions in other protein structures through the two sulfur atoms of the cysteine residues. Finally, LpMBD^core^ is terminated by a β‐strand E64‐V68 which interacts with the N‐terminal β‐strand. The two loops make close contacts through the hydrophobic side chains with an approximately orthogonal orientation between the loops. Interestingly, the CPXCGX‐containing loop displays considerable flexibility as deduced by comparison of an ensemble of the 10 best structural models. As such, the elucidated structure is in good agreement with the Alphafold prediction with an overall RMSD of 1.3 Å for Cα‐atoms (Fig. 1B).

### The metal‐bound structure of LpMBD and the trigonal sulfur‐based metal binding site

Next, we aimed to recover a copper‐bound structure of LpMBD^core^ to shed light on its functional role. Initial probing of metal binding to the previous employed LpMBD form by adding near‐stoichiometric amounts of copper caused precipitation in the sample, which could not be restored, and paramagnetic broadening of the signals. This was likely due to cycling of Cu between Cu^+^ and Cu^2+^ states, caused by a reduced redox potential of bound Cu^+^ to Cu^2+^ and the presence of 5 mm of the non‐chelating reducing agent tris(2‐carboxyethyl)phosphine (TCEP). Attempts to load Cu *in vivo*, even at low concentrations, resulted in death of the cultures, or indefinitely halting their growth. We therefore used the Cu^+^ congener Ag^+^ [43], which is compatible with standard liquid‐state NMR techniques, and not associated with the complications induced by copper. We followed the binding of Ag^+^ by gradually adding AgNO_3_ up to approximately equimolar amounts of the studied LpMBD form, observing chemical shift changes by ^15^N‐^1^H‐HSQC NMR (Figs S3 and S6). Significant conformational change would lead to shifting of peak resonances and can in some cases with exchange in the intermediate regime also cause line‐broadening beyond detection. During titration with Ag^+^ some peaks disappear due to line‐broadening at low added amounts of Ag^+^. These peaks correspond to residues involved in metal binding. At the final stages of titration, after adding equimolar or slightly excess amounts of Ag^+^, the exchange rate increases again, which causes almost all peaks to ultimately reappear at slightly perturbed positions. It is also observed that the unstructured form converts almost completely to the structured form as evident by the gradual disappearance of the peaks corresponding to the unstructured form (peaks marked with asterisks in Fig. S6A). The peaks corresponding to the unstructured segments only move little, but upon addition of equimolar amounts of Ag^+^, peaks for C18, H19, H20 and E21 broaden beyond detection. Following the last addition of Ag^+^, 3D experiments were recorded for resonance assignments and structural constraints as described above.

**Fig. 2:**
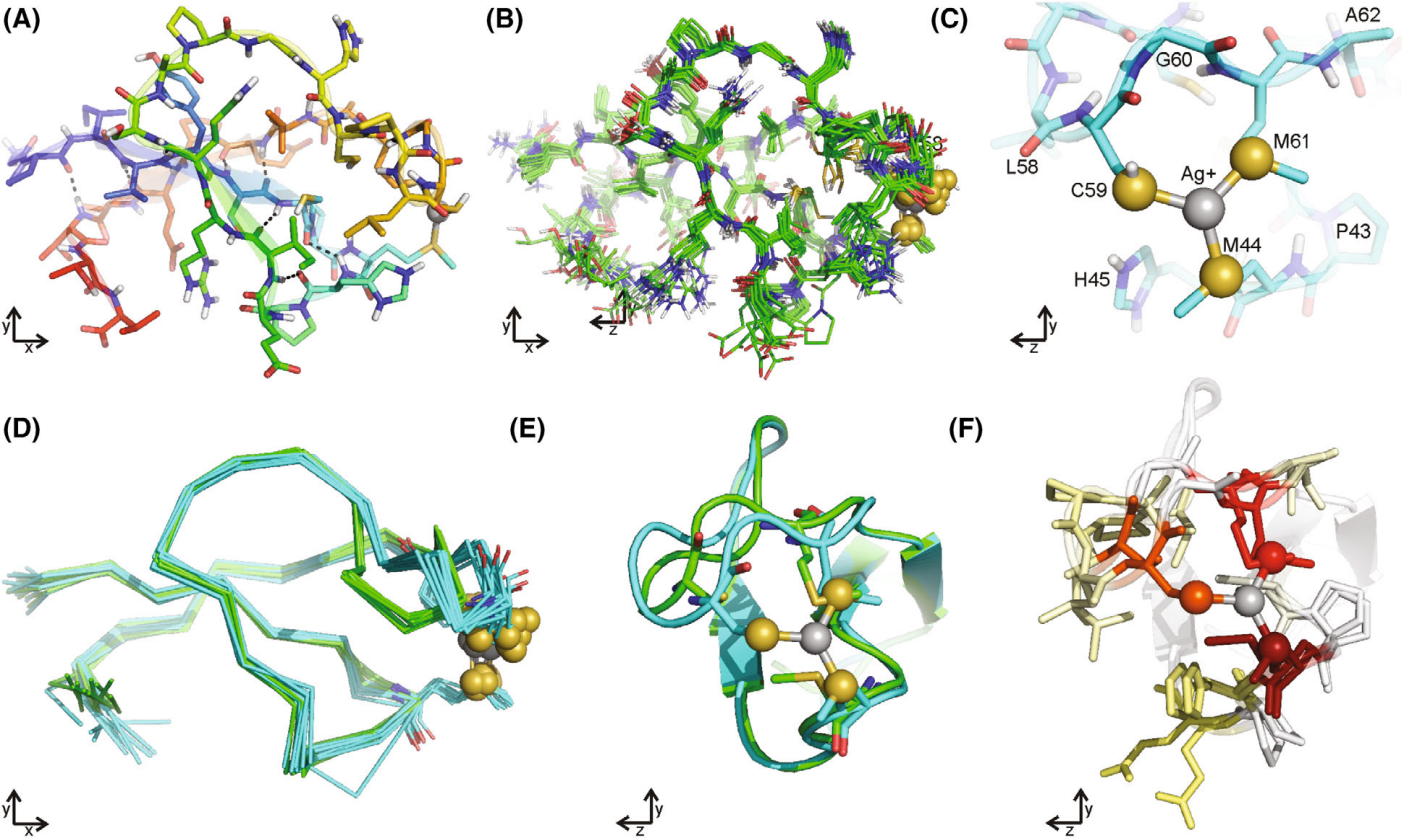
NMR structure of the Ag^+^‐bound form of the LpMBD^core^ and comparison with the apo form. (A) Cartoon representation showing Ag^+^ as a gray sphere. (B) Ensemble representation. (C) The metal binding site with residues closest to Ag^+^ annotated. (D) Ensemble overlay of the apo‐ and Ag^+^‐bound structures (green and cyan, respectively). (E) Overlay of the lowest energy apo‐ and Ag^+^‐bound structures (green and cyan, respectively). (F) Comparison of the Ag^+^‐bound and apo form, with each residue colored according to the difference in average chemical shift rms. See also Fig. S7. The panels with structures were generated using Pymol.

**Fig. 3:**
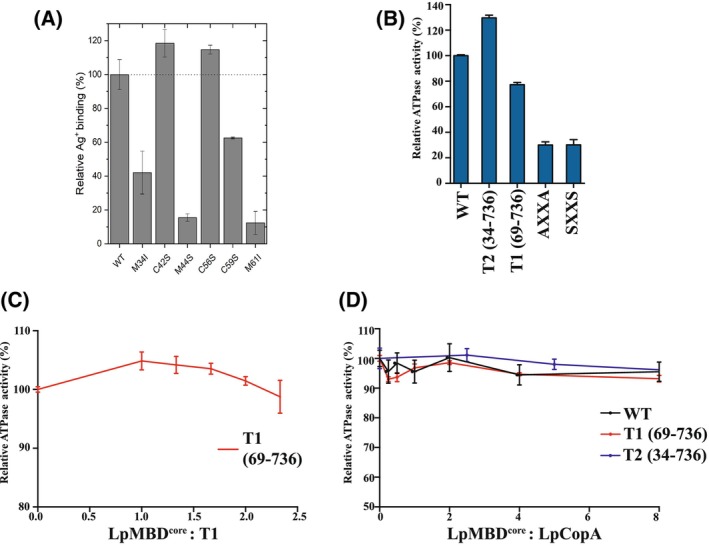
The function of the MBD and its N‐terminus. (A) Ag^+^ binding properties determined by ICP‐MS measurements of selected LpMBD mutants relative to wild‐type LpMBD (*n* = 2 for C42S, M44S, C56S, and C59S. *n* = 3 for WT, M34I and M61I). (B) Activity of LpCopA mutant forms compared to wild‐type (WT) protein. T1 and T2 denote truncations, see Fig. 1. AXXA and SXXS represent mutations of the so‐called CXXC motif of the MBD. The samples were purified from two different batches, the mean + SD of technical replicates is shown (*n* = 3). (C) Activity of the T1 truncation together with different molar ratios of the homology modeled core of the MBD:LpMBD^core^. (D) Activity of WT, T1, and T2 together with different molar ratios of the N‐terminal tail that precedes the LpMBD^core^:LpMBD^tail^. For all figures error bars are based on standard deviation over three technical replicates.

The overall shape of the metal‐bound structure is highly similar to the metal‐free form of LpMBD^core^ (Fig. 2). The largest structural differences between the apo and metal‐bound forms are detected for the two loops that are displaced 2 Å, while only small changes are detected for the β‐strand regions and the extended linker residues S51‐P53 (Fig. S3B). Specifically, the most significant shifts were observed for the sidechains of M44 and M61 where downfield ^13^C shifts have been demonstrated before to be caused by Ag^+^/Cu^+^ binding [44] (Fig. S3C). The two other methionine residues of LpMBD maintain their random coil‐like chemical shifts in the metal‐bound form. In addition, the sidechain of C59 shows a significant downfield ^13^C shift which is not seen for the other cysteine residues. Overall, the largest chemical shift changes cluster around M44, C59, and M61, both for the backbone and sidechain atoms (Fig. S3), which suggests they establish a tri‐sulfur coordination of Ag^+^ at the outermost tip where the two loops meet. The alternative sulfur ligands C42, C56 (the first cysteine of the CXXC motif) and M81 as well as the histidine sidechain nitrogens can be excluded due to the absence of any significant chemical shift change upon Ag^+^ addition. Interestingly, despite Hg^2+^ can bind to Ag^+^ sites in a non‐isostructural fashion, we observe a similar trend in the distribution of chemical shift changes when using Hg^2+^ for titration, further substantiating the nature of the metal coordinating ligands (Figs S6 and S8). To complement and validate the structures, we performed inductively coupled plasma‐mass spectrometry (ICP‐MS) measurements to quantify the relative Ag^+^ binding to mutant forms of the same MBD construct (M1‐R84) as used for the structural studies (Fig. 3A). Indeed, the reduced/abolished Ag^+^ binding relative to wild‐type determined by ICP‐MS measurements in selected isoleucine/serine mutants substantiates the role in metal binding by M44, C59, and M61 indicated by our NMR structures, while C42 and C56 are not participating in such interactions. Importantly, the binding mode of the LpMBD is reminiscent of the trigonal, exclusively sulfur‐coordinated binding mode of Cu^+^ in the LpCopA core as determined by EXAFS [45]. Thus, while in limited cases non‐isostructural Ag^+^ binding has been observed compared to Cu^+^ (primarily in Cu^+^‐cluster containing proteins, e.g. yeast Cu(I)‐metallothionein), the absence of multiple Cys‐rich motifs in the primary sequence, typical of Cu^+^‐cluster forming proteins, together with our NMR structures and binding data, suggest that Cu^+^ might be bound in a similar coordination mode as Ag^+^ in *Lp*CopA MBD, thereby acting as primary high‐affinity metal cargo over other transition metals (e.g. Zn(II)). Our results therefore indicate that the use of trigonal sulfur‐based coordination of the transported ion is maintained in LpCopA, and perhaps other Cu^+^‐ATPases all the way from initial binding to the MBD until ion release after translocation. The finding that LpMBD uses only C59 of the CXXC motif for metal ion coordination is somewhat surprising, as both sulfurs of this motif were originally considered to be necessary for proper coordination [30]. However, C59 may be important for other aspects of the metal handling, such as for ion delivery to the M‐domain of LpCopA.

### A conserved metal‐binding domain

To complement the structure of the metal‐bound form, we compared the structures to available models in the protein data bank (Fig. S9). Interestingly, the LpMBD was found to share overall fold with several zinc‐binding domains. They have in common the conserved cysteine residues aligning with C42, C56, and C59 and another cysteine/histidine, which aligns with H45 in the LpMBD. This could be relevant as H45 and C56 are located in the immediate vicinity of the M44, C59 and M61 site revealed by our NMR studies. This and the Hg^2+^‐binding data suggest that the metal binding site of the LpMBD^core^ structure is compatible with binding Zn^2+^ and other heavy metal ions, possibly indicating that in absence of Cu^+^/Ag^+^, this site may be occupied by other more common metal ions as has been shown for other types of MBDs (Fig. S8) [46, 47].

Interestingly, as identified using SWISS‐MODEL, our structures also demonstrate similarity to the Ag^+^‐bound N‐terminus of the metal‐binding domain of SilB (SilB N‐MBD) [48, 49, 50, 51, 52] (Fig. S9A). Both possess a rubredoxin knuckle fold with a trigonal sulfur coordination site of the metal ion, with the major difference being the use of a methionine in case of SilB for Ag^+^ coordination instead of C59 in LpMBD. SilB is an adaptor protein component of tripartite SilCBA metal exporters belonging to the resistance‐nodulation‐cell division (RND) superfamily [48]. There, SilB N‐MBD has been proposed to act as a sensor that upon metal binding activates the C‐terminal metal binding site of SilB, which then transfers the cargo ions to the SilCBA complex that extrudes them from the bacterial cell [48]. Given the similarity of the LpMBD^core^ and SilB N‐MBD, it is possible that LpMBD performs a similar metal‐sensing function in the LpCopA system, with the Cu^+^‐sensing residues able to also transfer the metal to the ATPase core [45].

### The N‐terminus of LpCopA is likely autoregulatory

Previous truncation studies of LpCopA (T0, ΔM1‐V74) have indicated a critical role of LpMBD for the LpCopA activity [30]. To further investigate the functional and mechanistic roles of the identified LpMBD^tail^ and LpMBD^core^ features, two alternative truncation forms (T1, 69–736; and T2, 34–736; Fig. 1B) were cloned, overproduced, and purified (Fig. S10). Their biochemical activity was then studied using an established (‘Baginski’) activity assay that monitors ion‐stimulated ATPase activity in lipid‐detergent micelles through detection of released inorganic phosphate [53] (Materials and Methods). Surprisingly, in contrast to the results previously obtained for T0, the T1, and T2 constructs displayed nearly wild‐type (WT) LpCopA activity, with a reduction of only ~20% for T1, and even a ~20% increase for T2 (Fig. 3B). Thus, the LpMBD does not appear to be critical for the transport mechanism, and the additional activity of the T2 construct rather hints at an autoinhibitory effect of LpMBD^tail^ (providing a negative effect on turnover that is removed in T2). This corroborates with the regulatory role of the MBD proposed for the well‐studied AfCopA [35]. The previously observed detrimental effect of the T0 truncation is likely a serendipitous event either linked to a non‐native structural arrangement caused by an unfortunate construct design or functional impairment by the N‐terminal His‐tag used for T0 [30] (Fig. 1B). The negative effect of such a tag on the activity was confirmed also for T2 (Fig. S10). Similar effects may explain the somewhat conflicting data on the role of MBDs for P_1B_‐ATPases [30, 32]. To validate the hypothesis that the N‐terminus of LpCopA is not involved in ion transport, we then explored whether the LpMBD^core^ can replace cellular chaperones in providing ion delivery to a MBD‐free ATPase core, in a manner similar to what has been demonstrated for ATP7B earlier [36]. To this end, we assessed the function of T1 LpCopA form in the presence of LpMBD^core^. However, the activity of T1 was unaltered over a range of LpMBD^core^ concentrations, supporting the notion that the N‐terminus does not have a critical role for providing ion delivery (Fig. 3C).

Notably, autoinhibitory mechanisms based on use of the unstructured segment of the N‐termini and/or certain of the loops that flank the MBDs (rather than the MBDs themselves) have recently been proposed for human ATP7B [36, 54, 55], which provides further support for the autoinhibitory role of the LpMBD^tail^. Thus, to shed light on the role of the LpMBD^tail^, we explored how WT, T1, and T2 were affected by *in vitro* synthesized LpMBD^tail^. However, the protein activity remained essentially unchanged for all three forms using different concentrations of LpMBD^tail^ (Fig. 3D). Thus, likely due to low affinity, a covalent link to either the entire ATPase or the LpCopA^core^ may be required for the LpMBD^tail^‐induced autoinhibition, and it is likely that the LpMBD^core^ guides the LpCopA^tail^ for binding.

### The metal‐binding motif of the MBD may serve as a sensor

Next, to dissect the detailed role of LpMBD^core^ and in particular its metal‐binding motif, we functionally characterized two separate full‐length forms of LpCopA. We limited our manipulations to C57 and C59, the latter shown by our NMR structure to be directly involved in ion binding while the former may participate in ion transfer, generating AXXA and SXXS substitutions of the CXXC motif, respectively. The two forms showed a ~70% activity reduction compared to WT, presumably caused by inability of the LpMBD^core^ to bind Cu^+^ [56, 57] (Fig. 3B). Our interpretation of this observation is that the release of autoinhibition is impaired, indicating that the metal‐binding motif of LpMBD^core^ is used as a copper sensor for orchestration of autoinhibition. The most likely model is that parts of the LpMBD interact with the core of the ATPase in the absence of copper, preventing conformational changes and futile ATP hydrolysis. In the presence of the transported ion, the LpMBD would then be released from the ATPase core and allow turnover.

### Hypothetical E1‐E2 transport cycle dependent position of the MBD

**Fig. 4:**
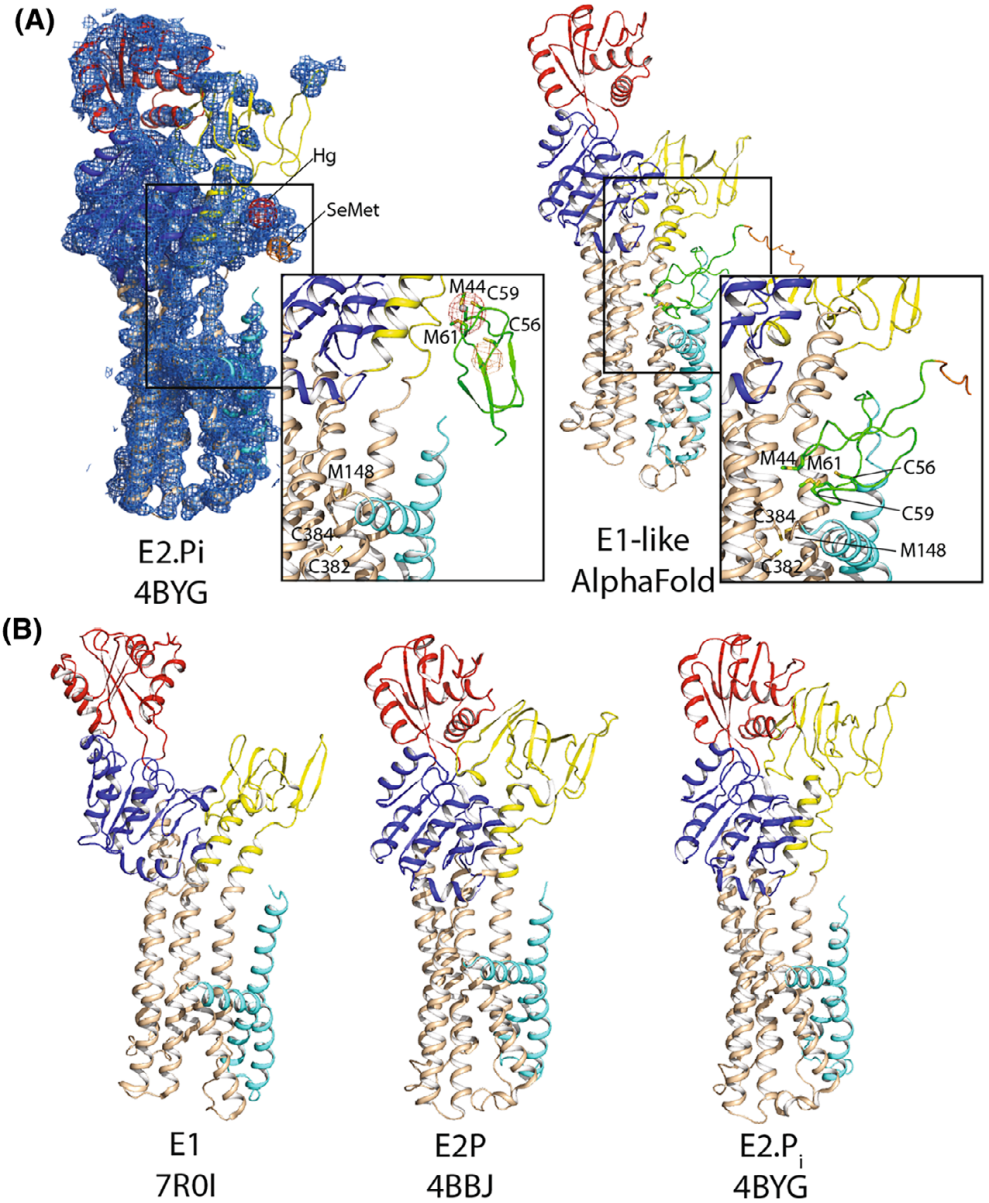
The transport mechanism dependent location of the core of the MBD of LpCopA. (A) Structure of LpCopA in the E2.P_i_ state (PDB: 4BYG) with the RESOLVE electron density (I/σ = 0.8) as well as anomalous difference Fourier peaks from selenomethionine‐derivatized or mercury‐soaked crystals (I/σ = 0.8) which were employed for putatively positioning LpMBD^core^. The inset shows the LpMBD^core^ NMR structure, together with the densities for Hg and SeMet. Key MBD residues are shown as sticks and labeled. For comparison the AlphaFold model is also included, with a similar inset for the position of the MBD. (B) The Alphafold generated model roughly represents an E1 state distinct to the available models of LpCopA in the E2.P_i_ and E2P conformations, as deduced through comparison with available structures of bacterial copper‐transporting P‐type ATPases. The position of the LpMBD^core^ in the E1 (deduced by Alphafold, Fig. S1) and E2.P_i_ states are not compatible with copper transfer to the entry site of the ATPase core. The panels with structures were generated using Pymol.

Except for a low‐resolution electron microscopy model which tentatively places the MBD of AfCopA between the A‐ and N‐domains in the E2.P_i_ transition state of dephosphorylation, little is known about the location of MBDs relative to the ATPase core in prokaryotic P_1B_‐ATPases, and possible state‐dependent positional variability [58]. However, indications of the position of the MBD relative to the ATPase core were detected in electron density maps exploited to generate the structure of the E2.P_i_ state of LpCopA [30]. Specifically, Hg and Se signals obtained from mercury soaks and selenomethionine‐incorporated sample, respectively, and an electron density map generated using density modification all indicated suggest adjacent to the A‐domain (Fig. 4A). These aspects were not part of the ATPase core and must hence be explained by the N‐terminus prior to V74 but were left unmodelled due to poorly resolved density. Now, with our NMR data at hand which also show that Hg^2+^ binding occurs at the same M44‐C59‐M61 site as Ag^+^, the signal corresponding to Hg^2+^ can be used as a reliable marker to more accurately positioning the LpMBD^core^ structure including its metal‐binding site (Fig. 4A). Thus, our findings indicate that the location of the LpMBD^core^ is adjacent to the A‐domain, and is in close proximity to N‐terminus of the ATPase core (V74) at least in the determined E2.P_i_ state, with the metal‐binding site facing the ATPase core. Yet, the original difficulties in modeling the LpMBD suggest the LpMBD^core^ is rather flexible in this step of the transport cycle.

In this context, it is relevant to assess the experimentally determined position with that observed in the Alphafold model. Unexpectedly, we identify that the latter largely represents an inward‐open E1 configuration, as deduced by comparison with the available E1, E2.P_i_ and E2P structures (root mean square deviations, RMSDs, of 3.4, 5.2 and 4.9 Å to PDB: 7R0I, 4BYG and 4BBJ), and through visual inspection of the model, in particular the domain arrangement that is reminiscent to that the E1 structure of AfCopA (Fig. 4B). Strikingly, Alphafold positions LpMBD^core^ on a feature in the membrane interface that connects transmembrane helices MB and M1, MB’, which has previously been suggested to be important for chaperone‐facilitated copper delivery, with the metal‐binding motif pointing towards the ion‐uptake region of the ATPase core and the co‐called entry site formed by M148, C382 and C384 [13, 30] (Fig. 4A). However, the residues of the entry site and the metal‐binding site of the LpMBD^core^ of the Alphafold model are not within distance for copper delivery, with the closest sulfur–sulfur distance, 9.4 Å, being that in‐between M661 and M148. Collectively, it thus appears likely that LpMBD^core^ is conformation sensitive in an E1‐E2 dependent manner and the indicated role of LpMBD^core^ is not contradicted by our structural analysis.

### A MBD‐ and conformation‐dependent nanobody suggests the N‐terminal tail inhibits turnover in copper‐free conditions

**Fig. 5:**
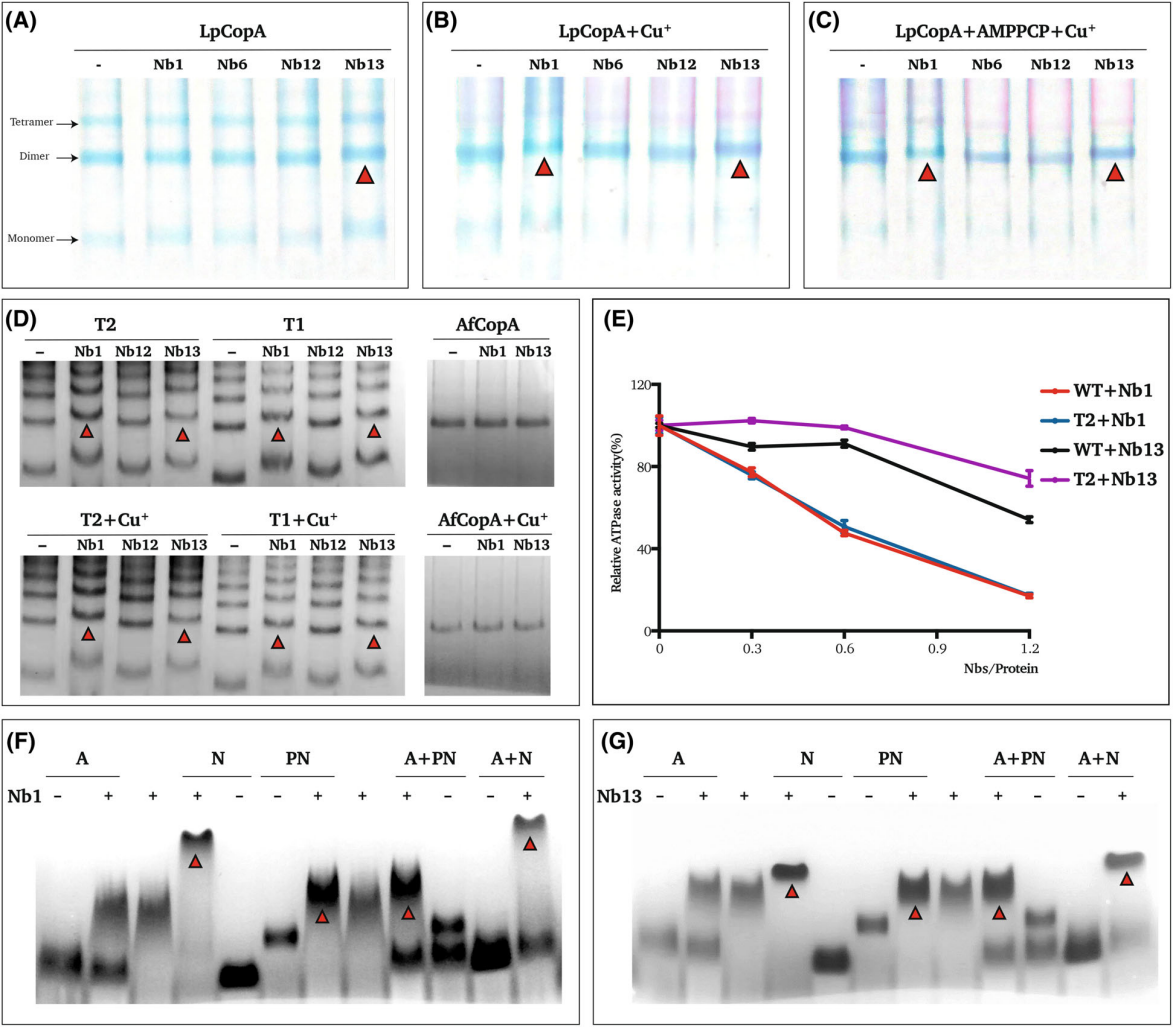
Identification of a MBD‐ and conformation‐dependent nanobody. All gels are native gels and complex formation is highlighted with red triangles, as determined by a shift in the target band in the presence of Nbs. Panels A–C represent Native PAGE Bis‐Tris gels that allow separation based on molecular weight only. Panels D and F, G are Novex Tris‐Glycine gels for which the separation is dependent on intrinsic charge and molecular size. (A–C) Initial binding assays to full‐length, wild‐type LpCopA (WT) of four different classes of nanobodies, Nb1, 6, 12, and 13 as well as a control without Nb (−), in three separate transport cycle intermediates. Conditions in the absence (A, for E2 states) or presence of copper (B, for early E1) or with copper and the nucleotide mimic AMPPCP (C, for late E1). The dominating LpCopA band, also without Nbs, appeared as dimers. Thus, Nb1 interacts in a copper‐dependent manner. (D) Analysis of Nb1 and Nb13 binding to MBD truncations of LpCopA (T1 and T2) and the homologous AfCopA (AfCopA‐T) in the presence and absence of copper. A control without Nb was employed (−). (E) Relative activity of WT and T2 in the presence of a range of molar ratios of Nb1 or Nb13. Error bars are based on standard deviation over three technical replicates. (F, G) Analysis of Nb1 and Nb13 binding to separately purified, isolated or mixed LpCopA soluble domains (A‐, P/N‐ and N‐domains). A control without Nb was employed (−). Nb1 and Nb13 associate with the N‐domains of LpCopA.

To identify a probe useful for validation of our hypothesis of separate functions of the core and tail of the MBD we turned to generation of nanobodies (Nbs). The intention was to identify a nanobody that binds to LpCopA in a MBD‐ and conformation‐dependent manner to investigate the regulation mechanism. A Nb‐library was generated based on llama immunization with purified detergent‐solubilized WT LpCopA over a period of 12 weeks with multiple injections. After two rounds of phage display followed by enzyme‐linked immunosorbent assays (ELISA), around 25 positive clones were identified. These were then reduced to 15 different Nb sequences based on redundancy. To map the interaction between WT LpCopA and isolated Nbs, we exploited native gels to assess complex formation, focusing on Nb1, Nb6, Nb12, and Nb13, thereby covering the most divergent nanobodies. To identify if the selected Nbs bound to LpCopA, we assessed three separate transport cycle states using conditions without and with copper, as well as with copper plus the nucleotide mimic AMPPCP. These conditions mimic the copper‐free E2, copper‐triggered early E1 or copper‐AMPPCP‐bound late E1 states, as previously demonstrated for structures of the P_2A_‐ATPase sarcoplasmic reticulum Ca^2+^‐ATPase (SERCA) [59, 60]. Interaction with LpCopA was detected in the absence of copper for Nb13, and in all conditions with copper for Nb1 and Nb13. In contrast, no/low‐affinity binding was observed for Nb6 and Nb12, precluding these targets from downstream efforts (Fig. 5A‐C), although it cannot be excluded that Nb6 or Nb12 associate in non‐tested states. Size‐exclusion chromatography was used to validate the binding (Fig. S11). Subsequently, to uncover if the binding of Nb1 and Nb13 is MBD‐dependent, we surveyed in a similar manner whether the interaction was maintained using the LpMBD^tail^‐free T1 and LpMBD^tail+core^‐free T2 forms of LpCopA. Strikingly, the two truncated variants now associated with both Nbs independently of copper concentration (Fig. 5D). As a control, Nb1 and Nb13 binding to a MBD‐truncated form of the homologous AfCopA (G80‐G736) was attempted, which revealed that these Nbs are highly specific to LpCopA (Fig. 5D).

Thus, MBD‐containing WT LpCopA associates with high affinity to Nb1 only in the presence of copper, whereas Nb13 interacts independently of copper concentration. In contrast, both Nb1 and Nb13 bind to T1 and T2 in a copper‐independent fashion. Nb1 hence displays the MBD‐ and conformation‐dependent LpCopA binding that we originally intended to identify using our Nb screening procedure. As only weak interactions between the LpCopA^core^ and soluble domains has been identified, our data therefore suggests that parts of the binding surface of Nb1 and LpCopA^tail^ is shared, as Nb1 can only bind LpCopA when binding of the LpCopA^tail^ has been released by presence of copper or truncation of the LpCopA^tail^. Moreover, Nb1 thus validates that LpMBD^tail^ binding and autoinhibition only occurs when copper is absent (in one or several E2 states or in an early E1 conformation). This is because Nb1 is unable to attach to full‐length LpCopA in the absence of copper, while it associates with T1 and T2 in a copper‐independent manner. As structures of full‐length LpCopA are available in the E2P and E2.P_i_ states without strongly defined features of the LpMBD, we propose that LpMBD^tail^ binding takes place in a late dephosphorylated E2 or an early E1 conformation in absence of Cu^+^.

### Nb1 inhibits LpCopA through interaction with the N‐domain

Considering the demonstrated functional effect of the LpMBD^tail^, and the predicted shared binding site for the LpMBD^tail^ and Nb1, it is likely that Nb1 inhibits LpCopA in a LpMBD^tail^‐like manner. To assess this, we again turned to the Baginski activity assay. We found that Nb1 profoundly reduces the activity of both WT and the T2 truncation, while the effect was less pronounced for Nb13 which was used as a control (Fig. 5E). We conclude that Nb1 is able to associate to LpCopA also in turnover conditions, likely stabilizing the same late E2 as LpCopA^tail^. Nb1 therefore also represents an attractive tool for downstream efforts to elucidate the structure of this elusive conformation of P_1B_‐ATPases, perhaps in combination with T1 or T2‐type truncations. To further explore how Nb1 (and to a lesser extent Nb13) reduces LpCopA activity, we investigated Nb binding to the soluble, isolated, A‐ (Q211‐Q333), N‐ (E433‐D553) and P/N‐domains (G405‐R662) of LpCopA, or combinations thereof, using a non‐denaturing electrophoretic mobility shift assay. This screening demonstrated that Nb1 and Nb13 both interact with the N‐ and P/N‐domains (Fig. 5F,G). This binding was also validated using size‐exclusion chromatography (Fig. S12). Nb1 therefore indeed behaves like the LpMBD^tail^ both in terms of its binding site and functional effects, likely reducing LpCopA transport by sterically interfering at the A‐/N‐domain interface, similarly to what was recently proposed for the human Cu^+^‐ATPase ATP7B [36], P4‐ and P5B‐ATPases [24, 25].

## Conclusions

**Fig. 6:**
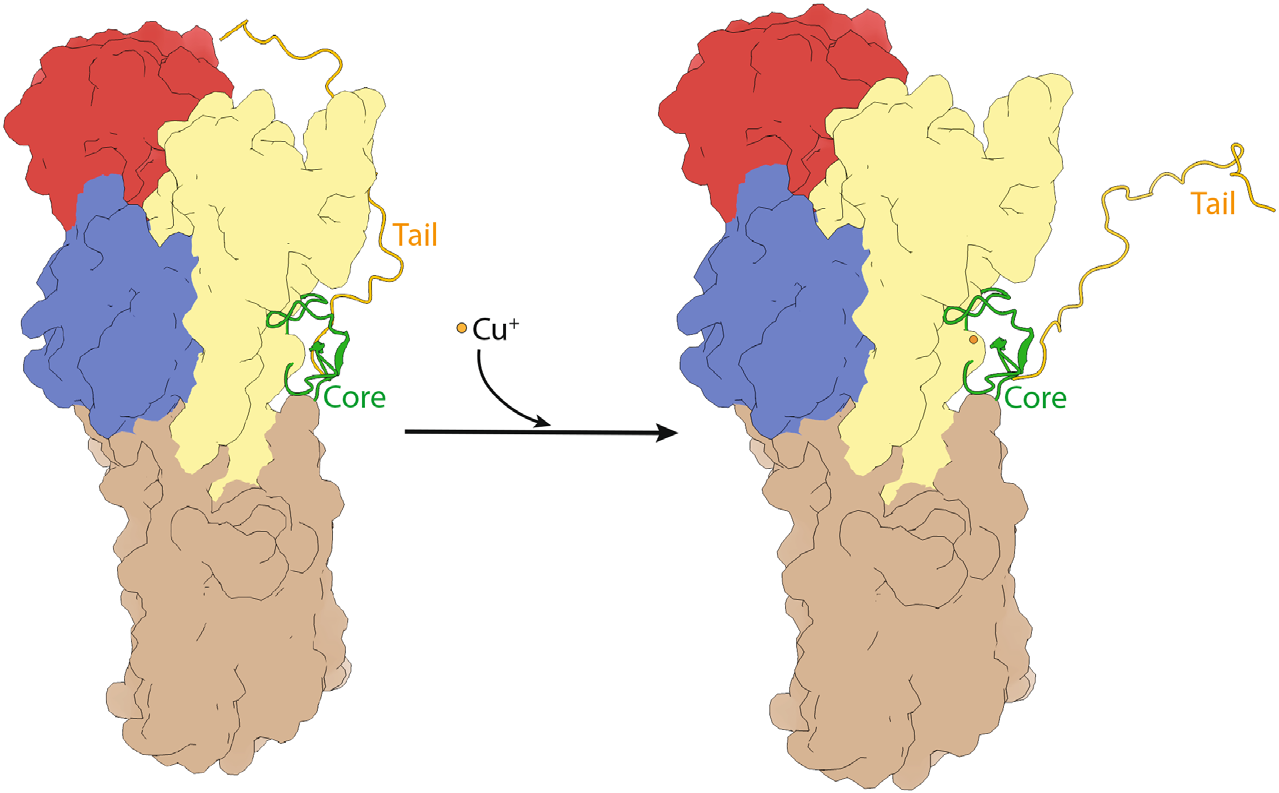
Proposed regulatory mechanism of the MBD. The N‐terminal LpMBD^tail^ that precedes the compact LpMBD^core^ binds between the soluble A‐ and N‐domains under normal cellular Cu^+^ levels and prevents turnover (see also Fig. 1A). The LpMBD^core^ serves as a sensor that signals to the LpMBD^tail^ when cytoplasmic concentrations of Cu^+^ (or Ag^+^) are elevated. The LpCopA^tail^ is then released from the ATPase core, permitting the necessary conformational changes to achieve ATP‐dependent efflux of Cu^+^/Ag^+^. When normal copper conditions are re‐established, the LpMBD^tail^ can bind again and re‐establish autoinhibition. A benefit of this regulatory setup is that the ion activation sensitivity of the ATPase core is decreased, which would prevent it from being active at normal cellular Cu^+^ levels due to its very high affinity to its translocated substrates.

Taken together, we propose a regulatory mechanism for the MBD of LpCopA (Fig. 6) in which the N‐terminal LpMBD^tail^ binds between the soluble A‐ and N‐domains of LpCopA under normal conditions, preventing enzyme turnover. The compact LpMBD^core^ serves as a sensor that signals to LpMBD^tail^ when cytoplasmic concentrations of Cu^+^ (or Ag^+^) are elevated. The LpMBD^tail^ is released from the ATPase core upon copper binding to the LpMBD^core^ in a sulfur‐coordinated trigonal fashion, permitting the necessary conformational changes to achieve ATP‐dependent ion efflux. When normal copper concentrations are re‐established, the LpMBD^tail^ will then bind again and re‐establish autoinhibition. The benefit of such a regulatory setup is that it decreases the sensitivity of these molecular pumps towards their translocated ions, which they otherwise bind with femtomolar affinity [61]. This prevents them from potentially starving cells of Cu^+^, an essential nutrient under normal conditions – yet allows quick activation of the pumps once needed, as Cu^+^ is dangerous to cells in excess.

## Materials and methods

Unless specified otherwise all chemicals and reagents were purchased from Sigma‐Aldrich (Burlington, MA, USA).

### Constructs

Full‐length wild‐type LpCopA was cloned into the vector pET‐22b(+) without a His‐tag, in contrast to previous efforts [30]. The truncations and soluble domains of LpCopA were PCR cloned into the same plasmid, employing an additional N‐terminal 8× His‐tag followed by the tobacco etch virus (TEV) protease cleavage sequence ENLYFQG (Fig. S10A). Mutants of SXXS and AXXA were achieved using the QuickChange procedure (Sigma‐Aldrich). The LpMBD^core^ construct was cloned based on T2 construct by adding one stop codon (mutating nucleotide C212A) in the C‐terminus of the LpMBD^core^. The LpMBD^tail^ was synthesized by the company OligoMaker ApS (Copenhagen, Denmark). The nanobodies were also cloned into pET‐22b(+) but using an N‐terminal signal peptide, and a C‐terminal TEV cleavage site followed by a 6× His‐tag. The primers used are listed in Table S1.

### Sample preparation for NMR

A sequence‐verified construct of the LpMBD (residues 1–84) in a pET22b backbone was transformed into *E. coli* strain C41, and 10 mL of positive clones cultured at 37 °C in LB until OD_600_ = 1.0 was reached. This was then used to inoculate 6 L of M9 minimal medium prepared using ^15^N‐NH_4_Cl and ^13^C‐glucose. Once OD_600_ = 0.4 was reached, the cells were induced with 1 mm IPTG for 16 h at 20 °C, and then harvested by centrifugation for 15 min at 5000 ***g***. Cell pellets were resuspended in 20 mm Tris/HCl pH = 7.6, 200 mm KCl, 20% (v/v) glycerol at 4 °C (1 g cells per 5 mL buffer). Prior to cell breakage 5 mm of fresh β‐mercaptoethanol, 1× SigmaFAST EDTA‐free protease inhibitor tablet and 2 μg·mL^−1^ DNase I were supplemented. The cells were then disrupted by two consecutive 25 000 p.s.i. runs using a cooled high‐pressure homogenizer. Unbroken cells and cellular debris were removed by centrifugation at 20 000 ***g*** for 1 h. The supernatant was filtered (0.22 μm), 50 mm imidazole was added, and the sample was loaded onto a 5 mL HisTrap HP column (GE Healthcare, Freiburg, Baden‐Wurttemberg, Germany). The LpMBD was eluted by a gradient from 50 to 500 mm imidazole in the resuspension buffer. The eluted protein was desalted into 20 mm Tris/HCl pH = 7.6, 100 mm KCl, 5 mm β‐mercaptoethanol, and TEV protease was added to remove the labeled protein's 6× His‐tag for 16–18 h at 10 °C. The sample was then applied to a 5 mL HisTrap HP column to remove uncleaved proteins and His‐tagged TEV protease. The sample was finally desalted into 10 mm MES pH 6.0, 100 mm KCl, 5 mm TCEP‐HCl. The ^15^N‐,^13^C‐labeled sample was then concentrated to ~100 mg·mL^−1^ (or 12.56 mm) using a VivaSpin concentrator (molecular weight cutoff, MWCO = 10 kDa). For titration experiments, up to roughly equimolar amounts of AgNO_3_ or HgCl_2_ were added. Titration experiments with CuNO_3_ in an oxygen‐free glovebox were unsuccessful, as they resulted in protein precipitation. Using CuCl_2_ in the presence of the non‐chelating reducing agent TCEP also resulted in eventual loss of the sample.

### NMR data analysis

Inspection of the acquired ^15^N‐^1^H heteronuclear single quantum correlation (HSQC) spectrum revealed more than the expected one peak per backbone and side‐chain amide groups, and contained both well‐dispersed and clustered resonances, which are respectively indicative of structured and unstructured proteins (Fig. S4A). The resonances were assigned using standard procedures and the GAMES_ASSIGN [62] software based on a series of different 3D multinuclear NMR experiments (NMR acquisition parameters and NMR resonance assignments, below). A subset of the peaks was not assigned initially, but further analysis revealed that these correspond to the LpMBD in an alternative unstructured conformation. The chemical shifts of the two forms only differ for residues G36‐V73, with most significant differences for P37‐T67 (Fig. S4B). The resonances for the metal‐bound form were assigned by the same procedure based on a set of 3D NMR experiments acquired separately.

Assigned chemical shifts are sensitive probes of secondary and tertiary structure and dynamics. Here we used the chemical shift‐based software NCSP [63] to infer the structured regions and discriminate between extended/sheet and helical/turn structure and RCI [64] to predict the degree of flexibility in terms of the so‐called order parameter, S^2^ (where values of 0 and 1 indicate completely flexible and rigid, respectively) and Talos‐N to derive ranges for backbone dihedral angles. This analysis reveals that (i) residues G36‐V73 have different chemical shifts for the metal‐free and metal‐bound forms. The segment with the smallest resonance dispersion (the *U*‐form henceforth, green curve in Fig. S4B) is predicted to be completely unstructured, whereas the one with larger resonance dispersion (the *S*‐form henceforth, black curve in Fig. S4B) is predicted to be structured for residues P37‐T67 as judged by S^2^ > 0.5. (ii) Three small beta‐strands are predicted for residues I38‐T41, R49‐Q50 and possibly T67‐V68 and two loops connect the sheets. (iii) The N‐terminal segment G1‐E35 containing 9 histidines and the C‐terminal segment V74‐R84, having degenerate chemical shifts for the two forms, are unstructured and dynamic (Fig. S4B) and give rise to the most intense peaks in the correlation spectra. There is an indication by NCSP of a transient/partial formation of a helical fragment for residues P76‐R84 (also mirrored in the Talos‐N predicted dihedral angles), which is in line with these residues being part of an alpha‐helix in the crystal structure. (iv) A flexible linker for residues T69‐V73, with very small differences in chemical shifts between the *U*‐ and *S*‐forms, bridges the structured core and the dynamic helical part in the *S*‐form.

We chose here to focus on structural characterization of the central structured region of the *S*‐form and therefore modeled residues G36‐V70. The structure was calculated using distance constraints derived from 3D ^13^C‐HSQC‐NOESY and ^15^N‐HSQC‐NOESY spectra and dihedral angle constraints derived from the chemical shift using TALOS‐N (see Structure calculation, below). NOESY signals corresponding to the structured form were assigned to ambiguous atom pair distance constraints using in‐house software. The structure was derived using a combination of Cyana and Xplor‐NIH combining data from both the metal‐free and the metal‐bound form (see Structure calculation below). Finally, the 10 best structural models being most consistent with the experimental data were chosen based on combined force field energy, distance/dihedral constraint energy, and agreement between observed chemical shifts and chemical shifts back‐calculated based on the structure using shiftX2 [65].

### NMR acquisition parameters

NMR data were collected on a Bruker 800 MHz AVANCE NMR spectrometer equipped with a z‐axis gradient, triple resonance cryoprobe and a Bruker 500 MHz AVANCE spectrometer equipped with a z‐axis gradient, triple resonance probe. Experiments were performed at 298 K. Heteronuclear 2D ^1^H–^15^N HSQC and ^1^H–^13^C HSQC and 3D HNCACB, HNCA, HNCO, HCCCONH, CCCONH, HCCH‐TOCSY (mixing time 10.9 ms) and both ^13^C‐ and ^15^N‐edited NOESY (mixing time 120 ms and 200 ms, respectively) experiments were performed on ^15^N,^13^C‐isotope labeled MBD in the apo form. Heteronuclear 2D ^1^H–^15^N HSQC experiments were performed to follow the titration of Hg/Ag into the sample and assignments of the metal‐bound form were confirmed using 3D HNCO, HCCCONH, and CCCONH experiments. All data were processed with TopSpin 3.2 (Bruker Biospin, Rheinstetten, Germany) and analyzed with Sparky.

### NMR resonance assignments

Total protein resonances were assigned using standard procedures based on heteronuclear 3‐dimensional NMR correlation spectra. In particular, peaks were grouped into dipeptide spin systems based on similarity of the H_N_ and N resonances, the spin systems were then assigned sequence specifically unambiguously using the software, GAMES_ASSIGN [62]. Finally, all remaining side‐chain resonances were assigned using the HCCH‐TOCSY spectrum in combination with the other spectra.

### Structure calculation

The structure of the structured core of MBD, residues G36‐V70 was calculated using distance constraints derived from 3D ^13^C‐HSQC‐NOESY and ^15^N‐HSQC‐NOESY spectra and dihedral angle constraints derived with TALOS‐N using the assigned chemical shifts. Since signals from the MBD were present for both the structured and unstructured forms, it was not possible to use standard automatic procedures for interpreting the NOESY data since due to the unstructured form peaks would risk being interpreted as a constraint for the structured form. Rather we used an in‐house developed software, Inferential Restraint Assignments (IRA, see below), to identify and leave out peaks which were likely to correspond to the unstructured form and used the remaining peaks to calculate the structure.

More specifically, all assignment possibilities consistent with chemical shifts were initially considered, except all cross peaks with assignment possibilities for residues in the unstructured form separated by four residues or fewer (commonly referred to as medium range). The remaining cross peaks were interpreted as ambiguous distance constraints and used to calculate a template structure ensemble. The peak assignments were then refined through four iterative cycles of NOE assignments and structure calculation, each time using this output structure ensemble as input for the NOE interpretation for the next cycle in a procedure similar to previously developed and widely used methods [66, 67]. The assignment candidates were ranked using our in‐house developed software, IRA, to estimate a probability, p, for each assignment possibility being consistent with peak position and assigned chemical shifts and the model structure ensemble. In each new round of assignments an increasing number of assignment possibilities with low estimated probabilities were disregarded leading to more and more unambiguous distance constraints (see Fig. S13 and Table S2).

NOE intensities, *I*, were translated into target distances, *d*, in constraints using *d* = *d*_max_(*I*/*I*_min_)^−1/6^ where *I*_min_ is the smallest intensity observed in the peak set and *d*_max_ = 4.25 Å were used as the maximum target distance. A square‐well potential was applied with a flat width of 0.33*d at both sides of the target distance.

We used Talos‐N [68] to predict dihedral angles φ and ψ for the backbone conformation. The predicted angles were used in the structure calculation if the prediction was classified as “Strong” by Talos‐N corresponding to a consistent prediction for a non‐dynamic residue. In the constraints, two times the spread in the Talos‐N prediction was used as the square‐well bound on both sides of the target dihedral angle.

Hydrogen bond constraints were added for residues predicted to be in β‐sheets with a probability >75%. These are residues I38‐T41 and R49‐Q50, which also have NCSP values indicative of β‐sheets. The hydrogen bond restraints were implemented as ambiguous distance constraints between H_N_ and C′ with a target distance of 1.7 ± 0.3 Å. For each hydrogen bonded residue, *i*, two ambiguous distance constraints were included to satisfy target distance between 1) H_N_(*i*)/C′(x) or H_N_(x)/C′(*i*−1) as well as 2) H_N_(*i*+1)/C′(x) or H_N_(x)/C′(i) (where “x” refers to any position in the sequence), corresponding to requiring that at least one of the edges of the β‐strand must be hydrogen bonded.

The chemical shifts were found to be very similar for the apo and bound form for the sheet and linker segments of MBD as measured by the weighted rms Δδ_rms_:

$$\begin{array}{l} \Delta \delta_{\mathrm{rms}}=\sum_{i=1}^{N}\frac{1}{n}\chi \left(\frac{\left(\delta_{i}^{\mathrm{apo}}-\delta_{i}^{\text{bound}}\right)}{\sigma_{i}},\tau_{i}\right),\\ \chi \left(x,\tau \right)=\left\{\begin{array}{c} x^{2}\;\text{if}\;x\geq 2\tau \\ \;0\;\text{if}\;x<2\tau \end{array}\right. \end{array}$$

where σ_*i*_ is the weight used to scale the chemical shift difference using 1.0, 10.0, and 4.0 for H, N, and C nuclei, respectively, and τ_*i*_ is the threshold for truncating the chemical shift difference using τ = 0.01977, 0.0176, 1 and 0.01780 for H_N_, N and C′, respectively and 0.02620 and 0.042974 for any other proton and carbon chemical shift, respectively. The individual thresholds were calculated from the standard deviation of the chemical shift difference for residues in the unstructured part of the LpMBD sequence displaying small fluctuations only in the chemical shift representing the precision of the measurement.

Accordingly, we decided to calculate the structure of both the apo‐ and the metal‐bound form simultaneously using a customized Xplor‐NIH structure calculation protocol, which keeps a specified part of the two structures superimposable. The superimposed parts were taken as residues P37‐T41, R49‐H55, and L63‐V68. Conversely, the first residue and the last two, which are the most dynamic, together with the two 7 residue loop segments C42‐I48 and C56‐A62, which host the metal ion were allowed to move freely. The similar structure segments were kept superimposed by using the Non‐Crystallographic Symmetry (NCS) potential in Xplor‐NIH. In addition, the Delphic torsions potential [69] was used to preserve proper local backbone geometry and the HBDB potential [70] was applied without specifying the hydrogen bonding partners to model favorable hydrogen bonding geometry.

Furthermore, constraints for the coordination geometry were imposed for the three Ag^+^ coordinating residues using 2.3 Å as the Ag‐S bond length, 120° as the S‐Ag‐S angle, and 109.5° as the Ag‐S‐C/H angle. 4000 structures were calculated and the 10 structures with lowest combined force field energy, distance/dihedral constraint energy and pseudo energy defined as the sum of the squared differences between observed chemical shifts and chemical shifts back‐calculated based on the structure using shiftX2. The structure statistics are shown in Table S2.

In the structure calculation in the first cycle of iterative NOE assignments and structure calculations, Cyana was used to calculate an initial model for both the apo‐ and Ag^+^‐bound form separately using standard parameters and NOE restraints assigned by IRA. Coordination of Ag^+^ was modeled implicitly using distance restraints between M61 and M44 Sε and C19 Sγ with a target distance of 4.0 Å. The lowest energy structures from this calculation were used as starting structure in the Xplor‐NIH structure refinement calculations and the Ag^+^ atom was placed initially in geometric center of the three coordinating *S* atoms.

### Inferential restraint assignments (IRA): Probabilistic ranking of NOE assignment possibilities

We assume that the probability of a certain assignment can be estimated as the product, P = *P*_*F*_ *P*_*D*_, of the probability, *P*_*F*_, for agreement between assignment and peak frequency and the probability, *P*_*F*_, that the two protons related to the assignments are close enough to produce a peak intensity above a certain threshold. We estimate the first probability as:

$$P_{F}=\frac{1}{\sqrt{2\pi }}\frac{1}{\sqrt{{\sigma_{F}}^{2}+{\gamma_{F}}^{2}}}e^{-\frac{1}{2}\Delta^{2}},\Delta^{2}=\frac{{\left\|\mu -a\right\|}^{2}}{\left({\sigma_{F}}^{2}+{\gamma_{F}}^{2}\right)}$$

where μ and *a* is the value of the resonance assignment and peak position, respectively, σ_*F*_ and γ_*F*_ is the corresponding uncertainties, and ||*|| denotes the normal Euclidian vector norm. Consider also that the intensity of a peak can be described by a log‐normal distribution centered around k*r ^−*n*^ with *n* = 6 and *r* being the corresponding distance which we assume to be log‐normal distributed around r_0_ which can be estimated from a template structure. Assume that statistically 50% of peaks will be observable if *r* < *r*_min_, *P*_*D*_ is estimated as:

$$P_{D}=k_{D}\Phi \left(\frac{n\left(r_{\min}-r_{0}\right)}{\sqrt{n^{2}{\sigma_{D}}^{2}+{\gamma_{D}}^{2}}}\right)$$

where Φ denotes the accumulative standard normal probability density function, while σ_*D*_ and γ_*D*_ are the uncertainties corresponding to log‐normal distributions for the distance and the peak intensity, respectively. Based on inspection of experimental data (not shown), we set γ_*D*_ = 1.0. If the template structure is an ensemble, the average of *P*_*F*_ among the models is used.

The structure of the metal‐binding domain was calculated through 5 iterative cycles of restraint assignments and structure calculations where some parameters above were kept fixed whereas others changed progressive during the cycles to allow for a gradual refinement of the structure. Throughout the calculations, we only considered assignments with reasonable chemical shifts agreement defined by *P*_*F*_ > 0.07. Furthermore, the standard deviation among observations of the same resonance within the spin system is used to estimate σ_*F*_, and γ_*F*_ = 0.2, 0.02, 0.3, and 0.05 ppm are used for N, H_N_, C, and H, respectively. In the initial phase where no model structure is available, we use conservative estimates for *P*_*D*_ = 1.0, 0.5, 0.3, 0.2, 0.15, and 0.1 for intra‐residue, sequential, residue difference (RD) = 2, RD = 3, RD = 4, and long‐range constraints, respectively. We used σ_*D*_ = 0.15 in the first 3 cycles but decreased it to 0.1 and 0.05 in the two last cycles of the procedure to reflect the increasing precision of the template structure. In each cycle, after the ranking of the assignments, assignment possibilities with a corresponding estimated probability, *P*_*i*_, were removed if *P*_*i*_ < *P*_max_/*f* where *P*_max_ is the maximum probability among the assignment candidates for this particular assignment and *f* > 1 is a factor which controls the extent of the assignment stripping. Here we use *f* = 50, 50, 20, 10, and 5 for the 5 consecutive cycles.

### Determination of Ag^+^‐binding to wild‐type LpMBD and mutant forms by inductively coupled plasma mass spectrometry (ICP‐MS)

Purified wild‐type LpMBD and mutant samples (500 μL; 5–20 μm) were titrated by addition of 3 Ag^+^ equivalents (from a AgNO_3_ stock in H_2_O) and incubated for 15 min at 18 °C under shaking. Protein samples were injected into a 5 mL HiTrap desalting column to remove unbound Ag^+^. The protein eluate aliquots (150 μL) were digested in concentrated HNO_3_ for 16 h. The mixture was diluted to 1.5 mL using Chelex®‐treated water (BioRad, Hercules, CA, USA). The Ag^+^ concentration was determined using ICP‐MS on a 7800 Inductively Coupled Plasma Mass Spectrometer (Agilent, Santa Clara, CA, USA), equipped with an autosampler. Prior to the analysis, Ag^+^ standards (Silver Standard for ICP‐MS by Sigma‐Aldrich) were prepared in 1% HNO_3_ to generate a calibration curve. Relative Ag^+^ concentrations were normalized to protein concentration based on integration of the of the chromatographic protein peaks (Abs_280_) and Ag^+^ content reported relative to wild‐type MBD.

### Protein purification for biochemical assays

Protein overproduction, cell disruption, membrane preparation, solubilization, and purification, using Ni‐NTA affinity resin and Superose 6 size‐exclusion chromatography, were performed as previously described for WT, T1, and T2 as well as a truncation of AfCopA [30]. The soluble domains of LpCopA (LpCopA^core^ (residues 34–70), A (211–333), N (433–553) and PN (405–662)), and nanobodies (Nb1, Nb6, Nb12 and Nb13) were generated as follows. Sequence‐verified constructs were transformed into C41 *E. coli*, and positive clones cultured at 37 °C in TB (Terrific Broth) medium until OD_600_ = 0.6 ~ 1 was reached. Cells were induced by supplementation of 1 mm IPTG (isopropyl β‐d‐1‐thiogalactopyranoside) for 16 h at 20 °C, and then harvested by centrifugation for 15 min at 5000 ***g***. Cell pellets were resuspended in buffer A (1 g cells per 5 mL buffer) containing 20 mm Tris/HCl, pH = 7.6, 200 mm KCl, 20% (v/v) glycerol through stirring at 4 °C for 2 h. Prior to cell breakage 5 mm of fresh β‐mercaptoethanol (BME), 5 mm MgCl_2_, 1 mm phenylmethylsulfonyl fluoride, 2 μg·mL^−1^ DNase I and Roche (Basel, Switzerland) protease inhibitor cocktail (1 tablet per 6 L cells) were supplemented. The cells were then disrupted by two consecutive 25 000 p.s.i. runs using a cooled high‐pressure homogenizer (Constant Systems, Daventry, UK). Unbroken cells and cellular debris were removed by centrifugation at 40 000 ***g*** for 1 h. The supernatant was filtered (0.22 μm) and loaded to 5 mL HiTrap Chelating HP columns (GE Healthcare, material from 6 L cells per column). Protein was eluted by a gradient from 0 to 500 mm imidazole in buffer C (20 mm Tris/HCl, pH = 7.6, 200 mm KCl, 1 mm MgCl_2_, 5 mm BME, and 20% (v/v) glycerol). Eluted protein was treated with TEV protease at a 10:1 molar ratio along with 1 mm EDTA and dialyzed in buffer E (20 mm Tris/HCl, pH = 7.6, 80 mm KCl, 1 mm MgCl_2_, 150 μg·mL^−1^ C_12_E_8_ (Nikko Chemicals, Tokyo, Japan), 5 mm BME, and 20% (v/v) glycerol) at 4 °C with gentle stirring for 16–18 h to remove the cleaved tag. The sample was then applied to a 5 mL HiTrap Chelating HP column (GE Healthcare) to remove uncleaved proteins and His‐tagged TEV protease. Subsequently, 10 mg of protein concentrated using VivaSpin devices (MWCO = 10 kDa for soluble proteins, MWCO = 50 kDa for WT, T1, T2, SXXS, AXXA) was subjected to size‐exclusion chromatography using a Superose 6 (GE Healthcare) column for truncations and mutations of LpCopA or a Superdex 75 column (GE Healthcare) for soluble LpCopA domains and the Nbs, each pre‐equilibrated with 50 mL buffer E. The eluted protein was analyzed using Coomassie‐stained SDS/PAGE and concentrated to approximately 10 mg·mL^−1^ using VivaSpin concentrators (MWCO as above), flash frozen in liquid nitrogen and stored at −80 °C until further use. Protein concentration was assessed using a Nanodrop spectrophotometer. SDS/PAGE showed that the protein samples were highly pure and the size‐exclusion chromatography that the preparations were homogeneous and suitable for downstream efforts (Fig. S10D).

### ATP hydrolysis assay

LpCopA forms were functionally characterized using the so‐called Baginski method [53]. Briefly, 15 μg of purified LpCopA was mixed with reaction buffer containing 40 mm MOPS‐KOH, pH = 6.8, 5 mm KCl, 5 mm MgCl_2_, 500 μm CuCl_2_, 20 mm cysteine, 150 mm NaCl, 3.0 mg·mL^−1^ C_12_E_8_ (Nikko Chemicals), 1.2 mg·mL^−1^ soybean lipids (Sigma‐Aldrich), 5 mm NaN_3_ and 0.25 mm Na_2_MoO_4_ in a total volume of 50 μL. For tests with supplementation of nanobodies or the LpMBD^core^, different domain concentrations were attempted at 500 μm CuCl_2_. The samples were then incubated at 37 °C with 500 rpm shaking for 5 min, and then added with 3 mm ATP (final concentration) to start the reaction and incubated at 37 °C with 500 rpm shaking for 15 min. 50 μL freshly prepared stop solution containing 2.5% (w/v) ascorbic acid, 0.4 m (v/v) HCl and 1% SDS was then supplemented to stop the reaction and start color development. 75 μL color solution (2% (w/v) arsenite, 2% (v/v) acetic acid, and 3.5% (w/v) sodium citrate) was added to the mixture following 10 min incubation at room temperature. Absorbance was measured at 860 nm following another 30 min incubation at room temperature.

### Analytical size‐exclusion chromatography and native PAGE

To monitor complex formation, the mixed proteins were incubated for 1 h and subsequently analyzed in duplicates using a Superose 6 (GE Healthcare) size‐exclusion column and/or native PAGE. As a control, the separate (non‐mixed) proteins were also sampled with 1 h incubation. All chromatography samples were run at 0.4 mL/min and monitored using UV absorbance at 280 nm. For native PAGE, 1000 mL 1× NativePAGE™ Anode Buffer was prepared using NativePAGE™ Running Buffer (20×), and 200 mL 1× NativePAGE™ Cathode Buffer was prepared using NativePAGE™ Running Buffer (20×) and NativePAGE™ 20× Cathode Additive. The samples were added NativePAGE™ Sample Buffer (4×) and NativePAGE™ 5% G‐250 Sample Additive before loading, and then loaded to 4–12% native gels (Thermo Scientific, Waltham, MA, USA). Novex Tris‐Glycine gels were used in which separation is based on intrinsic charge and molecular size. Electrophoresis was conducted at 150 V for 1 h, following which the voltage was changed to 250 V run for 30 min. The gels were each supplemented with 100 mL fixation solution (40% methanol, 10% acetic acid) and microwaved using ~1 kW for 45 s, and then placed on an orbital shaker for 15 min at room temperature. Subsequently, the fixation solution was decanted, 100 mL destaining solution (8% acetic acid) added, and the gels were microwaved using ~1 kW for 45 s. Finally, gels were shaken on an orbital shaker at room temperature until the desired background was obtained.

### Llama immunization and nanobody identification

Llama immunization and library generation was performed as previously reported, but now using a mixture of proteins including purified LpCopA for immunization [71]. The immunization was performed at Capralogics Inc. (Hardwick, MA, USA), which provides a healthy housing environment for all animals and adheres strictly to the United States Department of Agriculture Animal Welfare Act regulations for Animal Care and Use. Briefly, over a period of 12 weeks LpCopA (solubilized on 0.03% w/v DDM) were injected four times each with 100 g per injection. Ficoll paque plus (GE Healthcare, Life Sciences, Uppsala, Sweden) was used to isolate the peripheral blood mononuclear cells (PMBCs) and the total RNA were extracted using a RNeasy plus kit (Qiagen, Hilden, Germany). To generate the cDNA Superscript III first strand (Invitrogen, Thermo Fisher Scientific) was used and amplified using primers specific for the VHH genes. The PCR products were cloned into a phagemid vector that is designed to express Nbs as pIII fusions and with a C‐terminal E‐detection tag. A M13 phage‐display library were generated by use of VCSM13 helper phages. For the first round of selection 20 μg of biotinylated LpCopA (solubilized in 0.015% w/v C_12_E_8_) were bound to streptavidin beads and blocked in buffer E (supplemented with 2% w/v bovine serum albumin (BSA)) for 30 min. 5 × 10^13^ M13 phage particles were then incubated with the protein for 1 h followed by extensive washing with SEC buffer (15 times). The bound phage particles were eluted by incubation with 500 μL of 0.2 m glycine (Sigma‐Aldrich) pH 2.2 for 10 min, which then were neutralized by addition of 75 μL Tris pH 9.1 before it was mixed with 500 μL *E. coli* ER2738 cells. Before plating on agar plates (with 2% w/v glucose) the cells were incubated for 1 h at 37 °C. This enriched library was then used for a second round of phage display, but this time with only 1 μg of LpCopA and 2 × 10^12^ M13 phage particles to make it more stringent. In the last round of screening the expression of the nanobody and its ability to bind to LpCopA were assessed using ELISA. Single colonies were transferred to a 96‐well plate and grown for 4 h in LB medium, before the production of Nbs was induced by addition of 0.8 mm IPTG. Next day the plate was centrifuged and 50 μL of the supernatant was transferred to an ELISA plate coated with a total of 50 g LpCopA in SEC buffer blocked with 2% w/v BSA. After incubation for 1 h, the plate was washed four times with SEC buffer (without BME), and the anti‐E‐tag‐HPR antibody (Bethyl Laboratories Inc., Montgomery, TX, USA) was added followed by another incubation for 1 h. The plate was then washed four times in buffer E (without BME) followed by the addition of 50 μL 3,3′,5,5′‐tetramethyl‐benzidine (TMB, Sigma‐Aldrich). The reaction was quenched by addition of 50 μL of 1 m HCl and absorbance was measured at 450 nm. Positive phagemids were sequenced and sub‐cloned in pET22, containing a PelB signal at the amino‐terminus for periplasmic secretion and a carboxyl‐terminus C‐terminal TEV cleavage site followed by a 6× His‐tag.

## Conflict of interest

The authors declare no conflict of interest.

## Author contributions

QH, OS, JTN, NCN, PN, and PG initiated the project. OS performed cloning, overproduction, purification of the LpMBD^core^ for NMR structural studies, as well as the metal titration experiments. DWJ, ASPS, and JTN executed the NMR experiments. JTN calculated the LpMBD^core^ structures. QH performed cloning, overproduction, purification and functional studies of LpCopA and nanobodies, assisted by PL and CG. CG, KRA, and NSL generated nanobodies. QH, OS, VB, CG, and JTN prepared the figures QH, OS, CG, JTN, and PG and contributed to identification of scientific problem, data analysis, interpretation and writing of the first draft. All authors commented on the manuscript.

### Peer review

The peer review history for this article is available at https://www.webofscience.com/api/gateway/wos/peer‐review/10.1111/febs.17330.

## Supporting information


**Fig. S1.** Alphafold confidence scores for the model of LpCopA.
**Fig. S2.** IUPRED disorder predictions for selected copper transporting P_1B_‐ATPases.
**Fig. S3.** Analysis of metal binding to LpMBD.
**Fig. S4.** Assigned NMR resonances for the LpMBD.
**Fig. S5.** Schematic representation of chemical shifts for residues 36–70 in the structured and unstructured form of the LpMBD.
**Fig. S6.** Titration of the LpMBD.
**Fig. S7.** Structural comparison of the apo‐ and Ag^+^‐bound form of the LpMBD.
**Fig. S8.** Chemical shifts of the Hg^2+^‐bound LpMBD.
**Fig. S9.** Comparison of the LpMBD^core^ structure with SilB and other structural homologs.
**Fig. S10.** Purification of LpCopA and nanobodies.
**Fig. S11.** Initial nanobody screening using size‐exclusion chromatography.
**Fig. S12.** Nanobody binding with three soluble domains of LpCopA.
**Fig. S13.** Distribution of non‐trivial distance constraints.
**Table S1.** Primers used in this study.
**Table S2.** Restraint and structure statistics for the apo‐ and Ag^+^‐bound LpMBD NMR structures.

## Data Availability

The atomic coordinates and NMR data of the MBD of *L. pneumophila* Lpg1024 (LpCopA) have been deposited to the Protein Data Bank (https://doi.org/10.2210/pdb8OVL/pdb) and Biological Magnetic Resonance Bank under accession codes PDB: 8OVL and 34 812 (https://bmrb.io/data_library/summary/index.php?bmrbId=34812).
