# Supplementary material for: Transition metal transporting P‐type ATPases: terminal metal‐binding domains serve as sensors for autoinhibitory tails
Source: FEBS J. 2024 Nov 28;292(7):1654–74. doi: 10.1111/febs.17330 (PMC11970713; doi:10.1111/febs.17330)
Supplement: Supplementary file 1 — Fig. S1. Alphafold confidence scores for the model of LpCopA. Fig. S2. IUPRED disorder predictions for selected copper transporting P1B‐ATPases. Fig. S3. Analysis of metal binding to LpMBD. Fig. S4. Assigned NMR resonances for the LpMBD. Fig. S5. Schematic representation of chemical shifts for residues 36–70 in the structured and unstructured form of the LpMBD. Fig. S6. Titration of the LpMBD. Fig. S7. Structural comparison of the apo‐ and Ag+‐bound form of the LpMBD. Fig. S8. Chemical shifts of the Hg2+‐bound LpMBD. Fig. S9. Comparison of the LpMBDcore structure with SilB and other structural homologs. Fig. S10. Purification of LpCopA and nanobodies. Fig. S11. Initial nanobody screening using size‐exclusion chromatography. Fig. S12. Nanobody binding with three soluble domains of LpCopA. Fig. S13. Distribution of non‐trivial distance constraints. Table S1. Primers used in this study. Table S2. Restraint and structure statistics for the apo‐ and Ag+‐bound LpMBD NMR structures. [file FEBS-292-1654-s001.pdf]

Supporting information

Transition metal transporting P-type ATPases: terminal metal-binding domains serve as sensors for autoinhibitory tails

Qiaoxia Hu[1], Oleg Sitsel[2,3], Viktoria Bågenholm[1], Christina Grønberg[1], Pin Lyu[1], Anna Sigrid Pii Svane[4], Kasper Røjkjær Andersen[2], Nick Stub Laursen[2], Gabriele Meloni[5], Poul Nissen[2], Dennis W. Juhl[4], Jakob Toudahl Nielsen[4], Niels Chr. Nielsen[4]† & Pontus Gourdon[1,6]†.

[1] Department of Biomedical Sciences, University of Copenhagen, Blegdamsvej 3B, 2200 Copenhagen N, Denmark.
[2] Department of Molecular Biology and Genetics, Aarhus University, Universitetsbyen 81, 8000 Aarhus C, Denmark.
[3] Current address: Marine Structural Biology Unit, Okinawa Institute of Science and Technology Graduate University, Onna, Okinawa 904-0495, Japan.
[4] Interdisciplinary Nanoscience Center (iNANO) and Department of Chemistry, Aarhus University, Gustav Wieds Vej 14, 8000 Aarhus C, Denmark
[5] Department of Chemistry and Biochemistry, The University of Texas at Dallas, 800 W Campbell Road, Richardson, TX 75080, USA.
[6] Department of Experimental Medical Science, Lund University, Sölvegatan 19, Lund, 221 84, Sweden.
† Correspondence: Niels Chr. Nielsen: ncn@chem.au.dk, +45 28 99 25 41, and Pontus Gourdon: pontus@sund.ku.dk, +45 503 399 90

Supplementary Figure 1

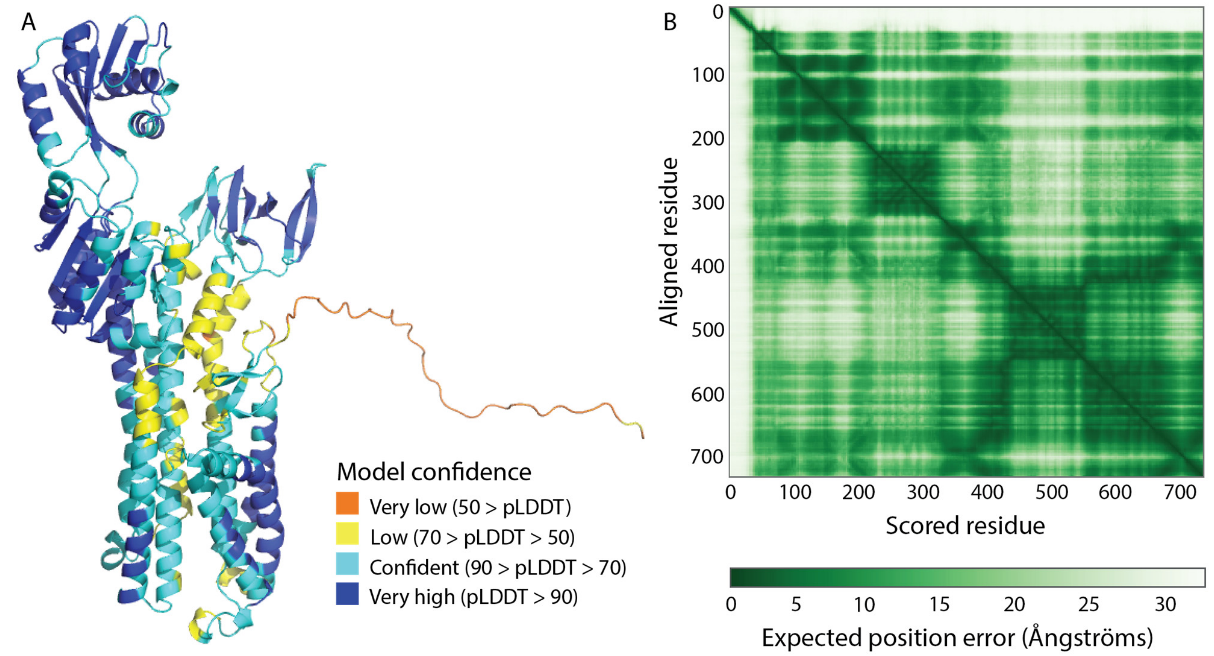

Supplementary Figure 1 | Alphafold confidence scores for the model of LpCopA. pLDDT scores and predicted aligned error (PAE) for the Alphafold model of LpCopA.

Supplementary Figure 2

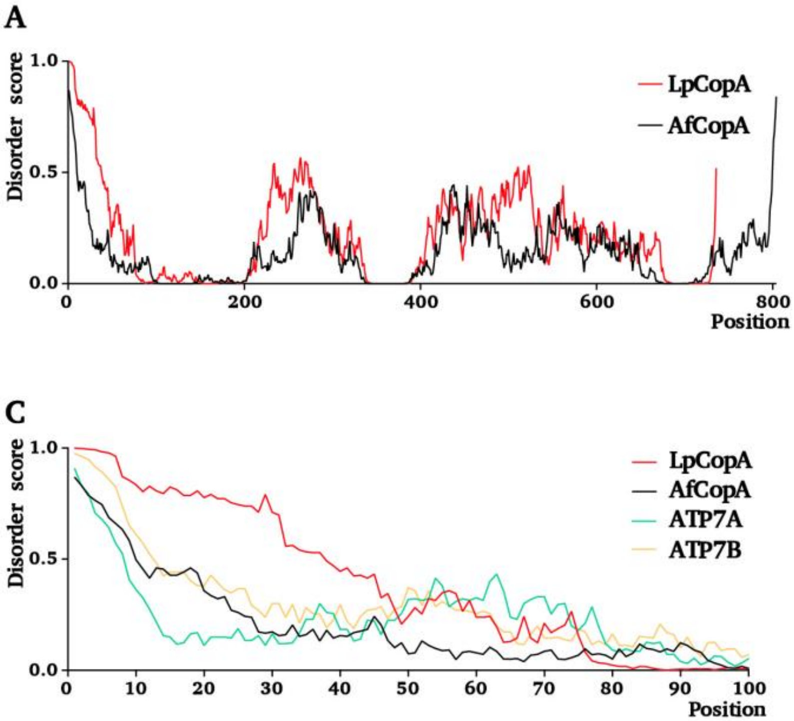

Supplementary Figure 2 | IUPRED disorder predictions for selected copper transporting P_{1B}-ATPases. A. The N-termini of LpCopA and AfCopA are predicted to be disordered. **B.** The disordered N-terminus of LpCopA is longer than that of AfCopA, ATP7A and ATP7B.

Supplementary Figure 3

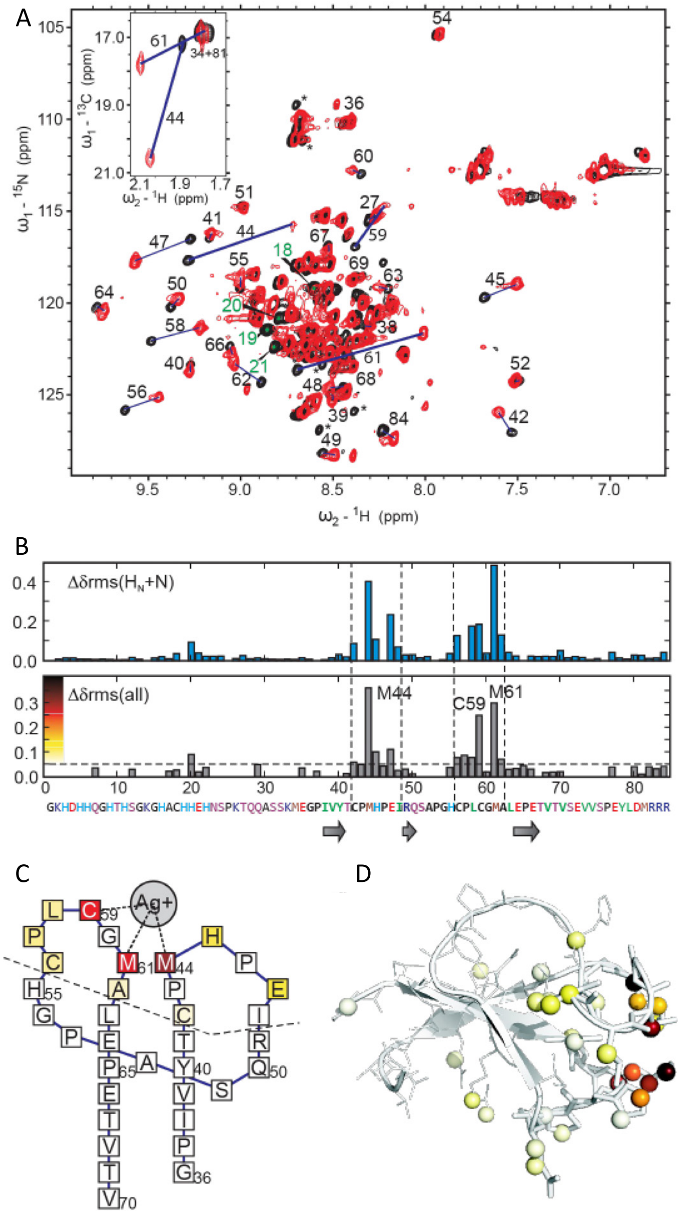

Supplementary Figure 3 │ Analysis of metal binding to LpMBD. A. Overlay of ^{1}H-^{15}N HSQC spectra for the apo- and Ag^{+}-bound form using black and red contours, respectively. Sequence specific assignments are shown on lines connecting the peaks for the two forms (see also **Supplementary Figure 5**). *Inset*: The methionine-rich region of the ^{1}H-^{13}C HSQC. **B.** Weighted rms chemical shift difference between the apo- and Ag^{+}-bound form. **C.** Visualization of all atom rms shown in B and **D.** visualization of the chemical shift rms grouped separately for each CH/CH_2/CH_3 group (color coding as in B).

Supplementary Figure 4

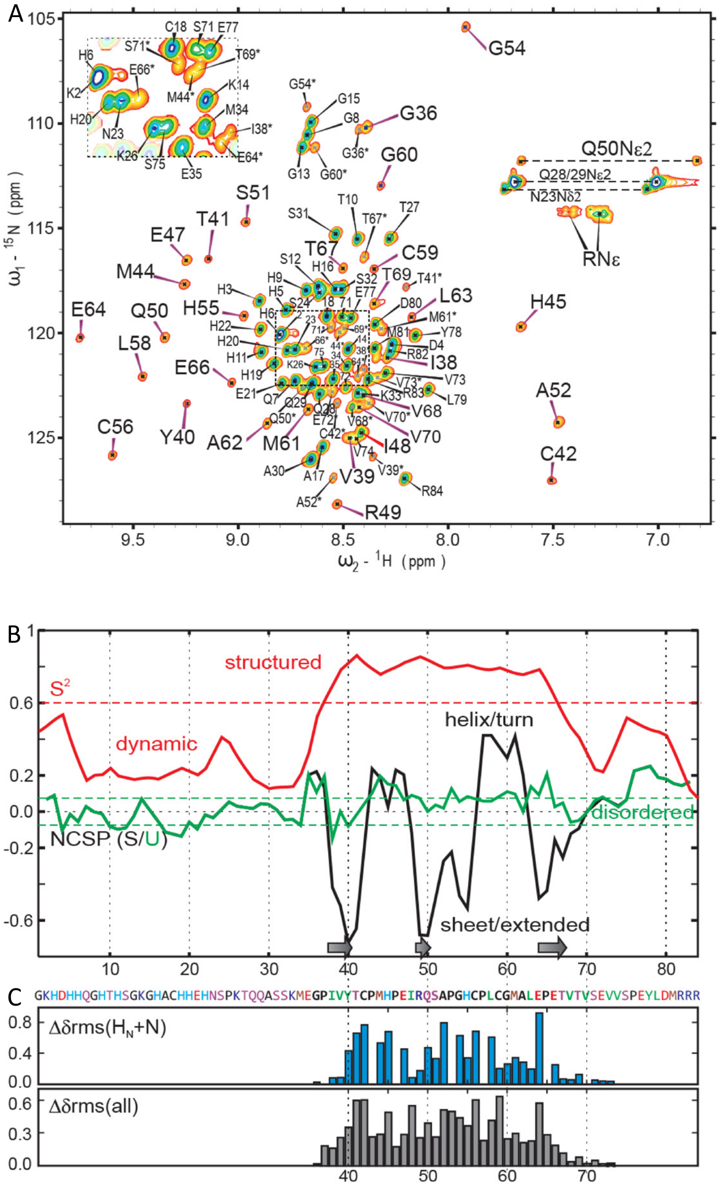

Supplementary Figure 4 │ Assigned NMR resonances for the LpMBD. A. H-^{15}N HSQC showing assigned peaks. Peaks corresponding to the structured core are showing in larger font size and with magenta wedges. Peaks corresponding to the minor unstructured form are marked with asterisks. B. Per residue chemical shift based predicted properties (see main text) NCSP for the *S*- and *U*-form shown with black and green curves, respectively, and S^2 for the S-form (red curve). C. Weighted chemical shift rms for differences between assigned chemical shifts for the *U*- and *S*-form, using 1/10 and 1/4 as weights for ^{15}N and ^{13}C shifts, respectively. H$_N$+N and all shifts (including side chains) are respectively shown as blue and grey bars.

Supplementary Figure 5

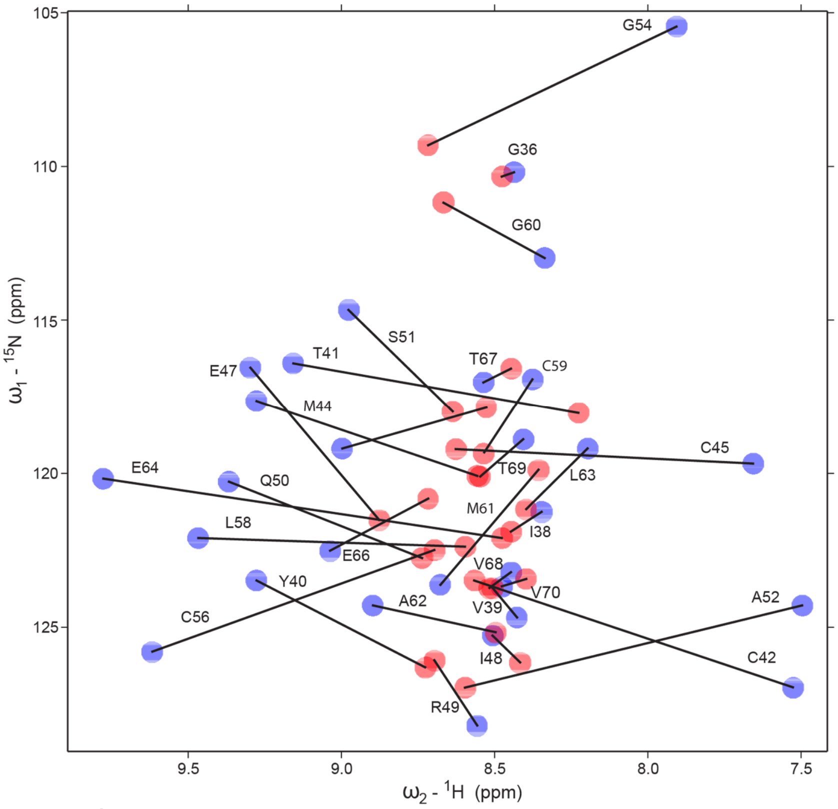

Supplementary Figure 5 | Schematic representation of chemical shifts for residues 36-70 in the structured and unstructured form of the LpMBD. Each pair of backbone H_N,N ^{1}H, ^{15}N chemical shifts are shown as blue and red disks for the structured and unstructured form, respectively. Chemical shifts for the same residue are connected by lines.

Supplementary Figure 6

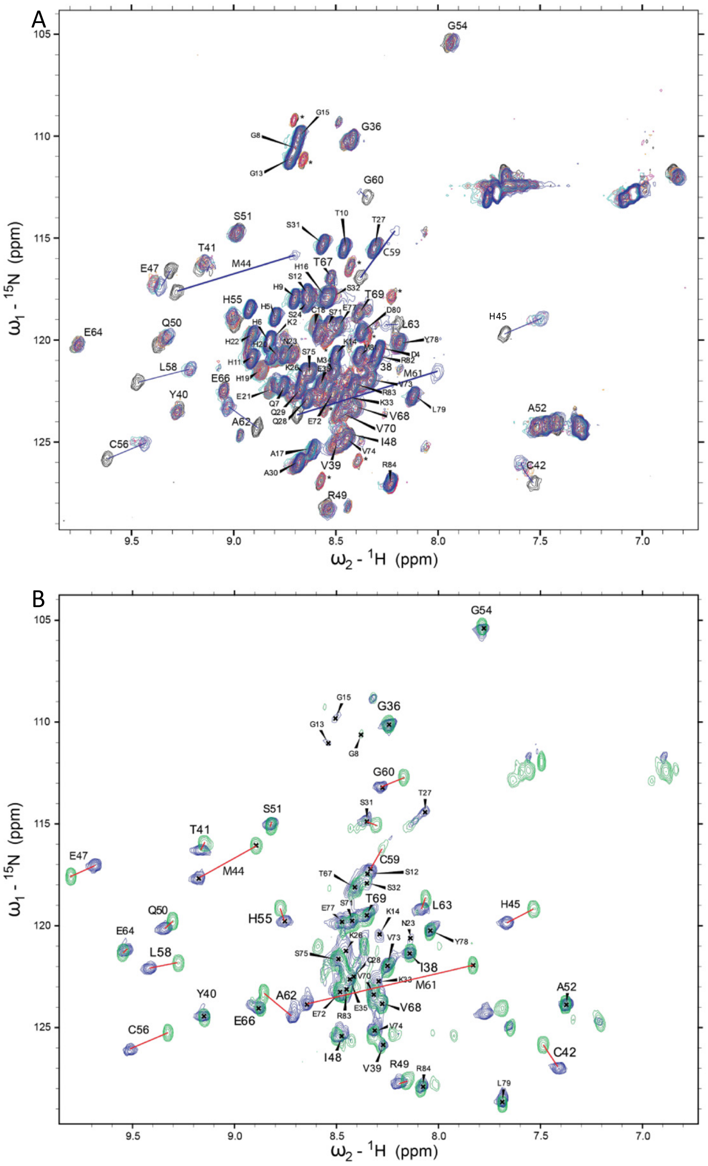

Supplementary Figure 6 │ Titration of the LpMBD. A. ^{1}H-^{15}N HSQC spectra of the LpMBD shown as black, orange, red, purple, cyan, and blue contours after the addition of increasing amounts of Ag^{+}. Peaks corresponding to starting and end-point titrations are connected by blue lines in case of significant chemical shift changes. Sequence specific assignments are given above the connecting line, near the peak or using a wedge. Peaks (corresponding to the unstructured minor form) which disappear at some point in the titration are marked with asterisks. **B.** ^{1}H-^{15}N HSQC spectra of LpMBD prepared using an alternative procedure (Materials and Methods) after adding equimolar amounts of Cu^{+} annotated as in A, with large chemical shift changes shown by red lines.

Supplementary Figure 7

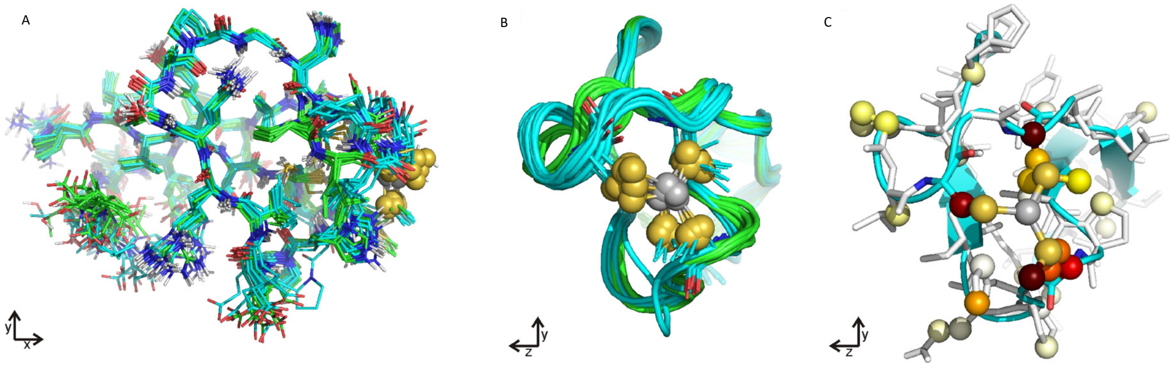

Supplementary Figure 7 │ Structural comparison of the apo- and Ag^{+}-bound form of the LpMBD. A. Overlay of the ensemble structures for the apo- and Ag^{+}-bound form shown with sticks. **B.** Cartoon representation, showing beta sheets as tubes. **C.** Cartoon representation of the Ag^{+}-bound form shown with a cyan backbone. White sticks indicate the two loops. Colored spheres are used for atoms with largest difference in chemical shifts between the metal-bound and apo forms.

Supplementary Figure 8

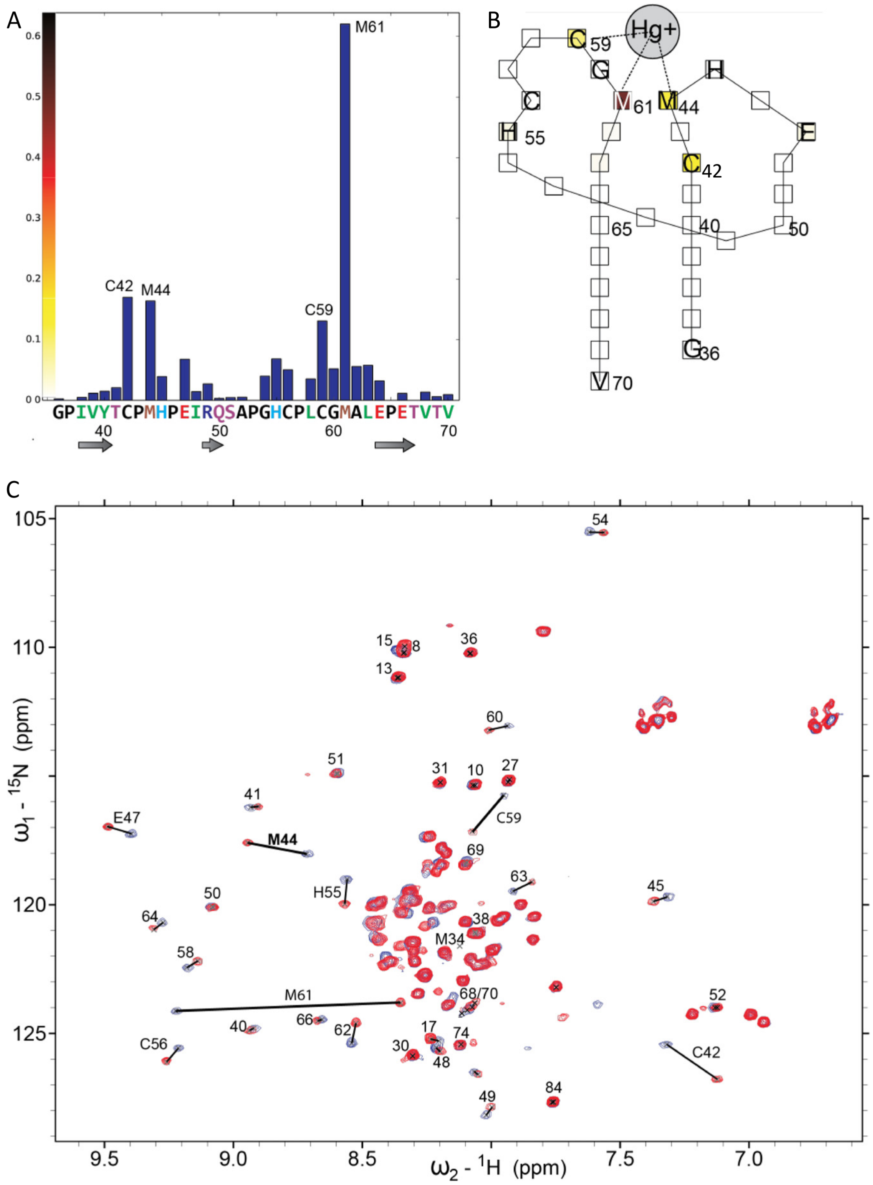

Supplementary Figure 8│ Chemical shifts of the Hg^{2+}-bound LpMBD. A. The weighted rms N,H_N chemical shift difference between the apo- and Hg^{2+}-bound form of the LpMBD. **B.** Visualization of the rms shown in A. **C.** Overlay of ^1H-^{15}N HSQC spectra of the LpMBD apo form (red contours) after the addition of equimolar amounts of Hg^{2+} (black contours). Peaks corresponding to starting and end-point titrations are connected by black lines in cases with significant chemical shift changes. Sequence specific assignments are given above the connecting line. The putative position of M34 is indicated in the spectrum by a small grey cross.

Supplementary Figure 9

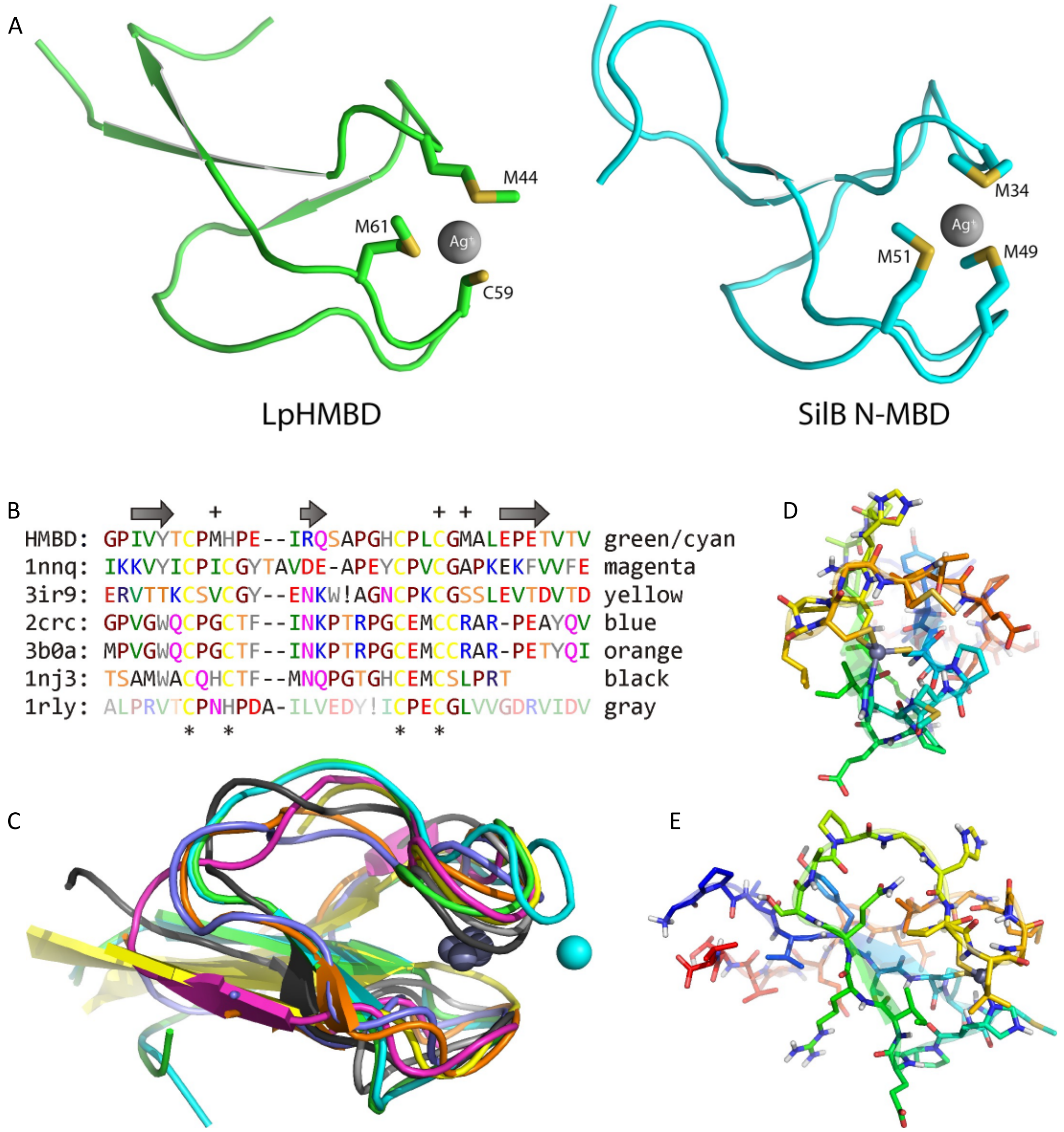

Supplementary Figure 9 │ Comparison of the LpMBD^{core} structure with SilB and other structural homologues. A. A comparison of the Ag^{+}-bound LpMBD^{core} and SilB N-MBD structures. Residues coordinating the silver ion are indicated. **B.** Sequence alignments showing segments of proteins structurally homologous to the LpMBD^{core}. The PDB IDs are shown to the left and color coding used in B is indicated to the right. The sequence alignment was adjusted manually for some residue positions to account for conservation in the structural alignment. β-sheet segments of the MBD are highlighted with arrows and the Ag^{+}-binding site with "+" in the top. Conserved Zn^{2+} coordinating residues Cys/His are highlighted in the bottom with asterisks. "!" indicate non-aligned

residues (8 and 5 residues for 3ir9 and 1rly, respectively). The PDB IDs correspond to the following proteins. 1nnq: rubrerythrin from *Pyrococcus furiosus* pfu-1210814. **3IR9**: C-terminal domain of peptide chain release factor from *Methanosarcina mazei*. **2CRC**: zf-ranbp domain of the HBV-associated factor from *Homo sapiens*. **3B0A**: HOIL1-L-NZF from *Mus musculus*. **1NJ3**: conserved NZF domain of Npl4 from *Rattus norvegicus*. 1rly: zinc ribbon domain of the general transcription factor TFIIB from *Homo sapiens*. **C.** Cartoon representation of protein segments from A, aligned using PyMOL. Zn^{2+} and Ag^{+} ions are shown as gray and cyan spheres, respectively. The reference structure of the $LpMBD^{core}$ from this work is shown in green and cyan for the apo- and metal-bound form, respectively. Non-aligned segments as indicated by "!" marks or shaded letters in a are not shown in the overlay to increase readability. **D, E.** Zn^{2+}-bound model built based on the Ag^{+}-bound structure and loose NOE-derived distance constraints coordinating Zn^{2+} to C42, H45, C56, and C59 as suggested by sequence alignments.

Supplementary Figure 10

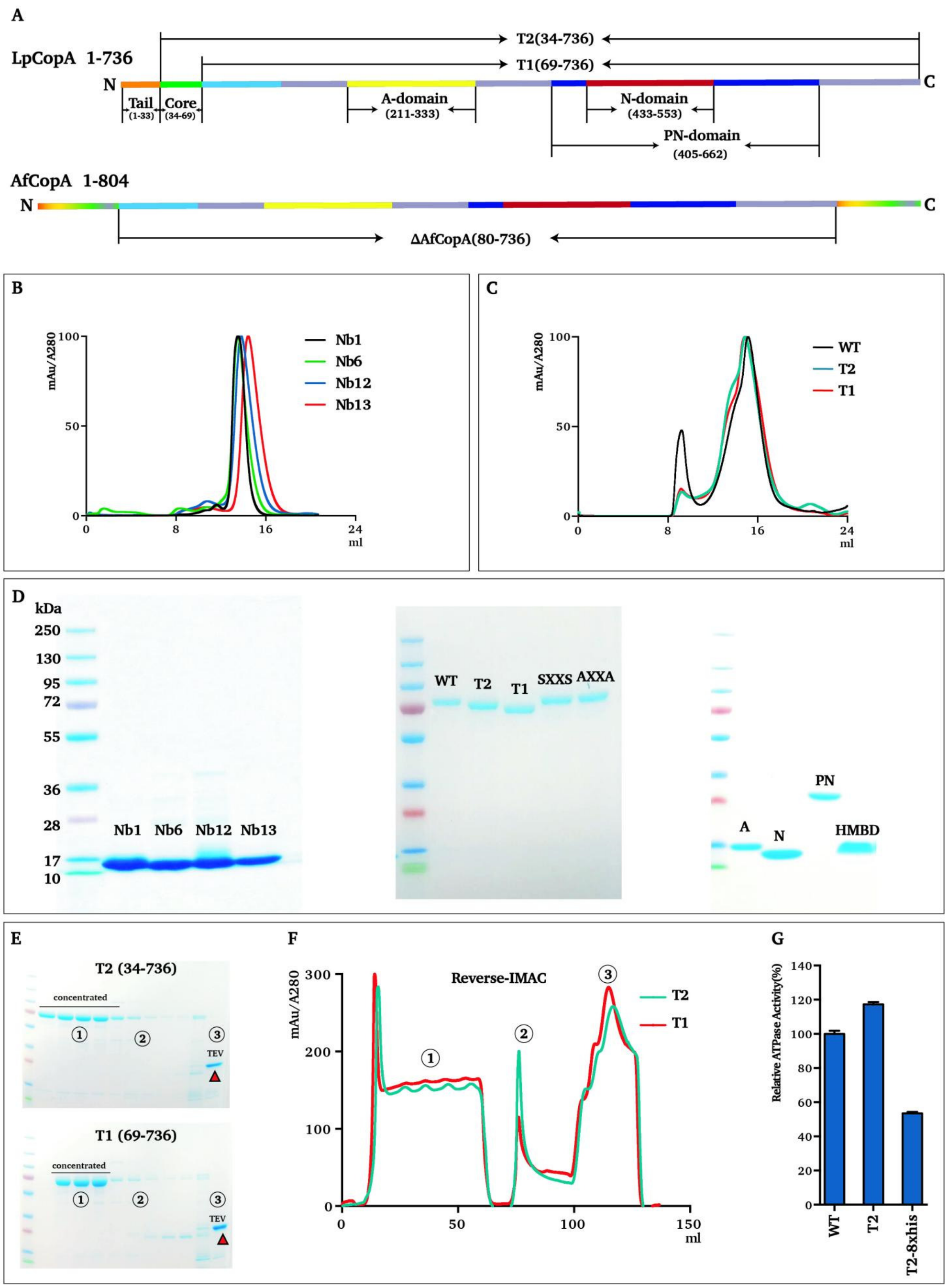

Supplementary Figure 10 │Purification of LpCopA and nanobodies. A. Overview of constructs used in this paper. The domains are colored as in Figure 1. Large scale purification size-exclusion profile of LpCopA (**B**) and nanobodies (**C**). The proteins were purified using Superose 6 (LpCopA) and Superdex 75 (nanobodies) size-exclusion chromatography columns and monitored by UV absorbance at 280 nm. **D.** SDS-PAGE of the eluted protein (nanobodies, WT and two truncations of LpCopA, four soluble domains of LpCopA, A, N, PN, and MBD) were highly pure based on

Coomassie-stained SDS-PAGE analysis. **E-G.** Purification of LpCopA forms and the effect of N-terminal His-tag**. F**. Reverse IMAC profiles of LpCopA truncations T1 and T2. The N-terminal His-tag was removed by TEV protease digestion using a molecular weight ratio of 10:1, in the presence of 1 mM EDTA. Following addition of 30 mM imidazole and sample filtration, the samples were loaded to an ÄKTA to run a reverse IMAC program. **E**. SDS-PAGE results of purification of T1 and T2 following reverse IMAC. The samples eluted in step 1 were highly pure and concentrated for size-exclusion chromatography, less target protein was eluted in steps 2 and 3, particularly in step 3. **G.** Activity of LpCopA and its T2 truncation with or without His-tag, tested using the Baginski assay. T2 with an 8x His-tag at the N-terminus has ~60 % less activity compared to T2 without a His-tag. Error bars are based on standard deviation over three technical replicates.

Supplementary Figure 11

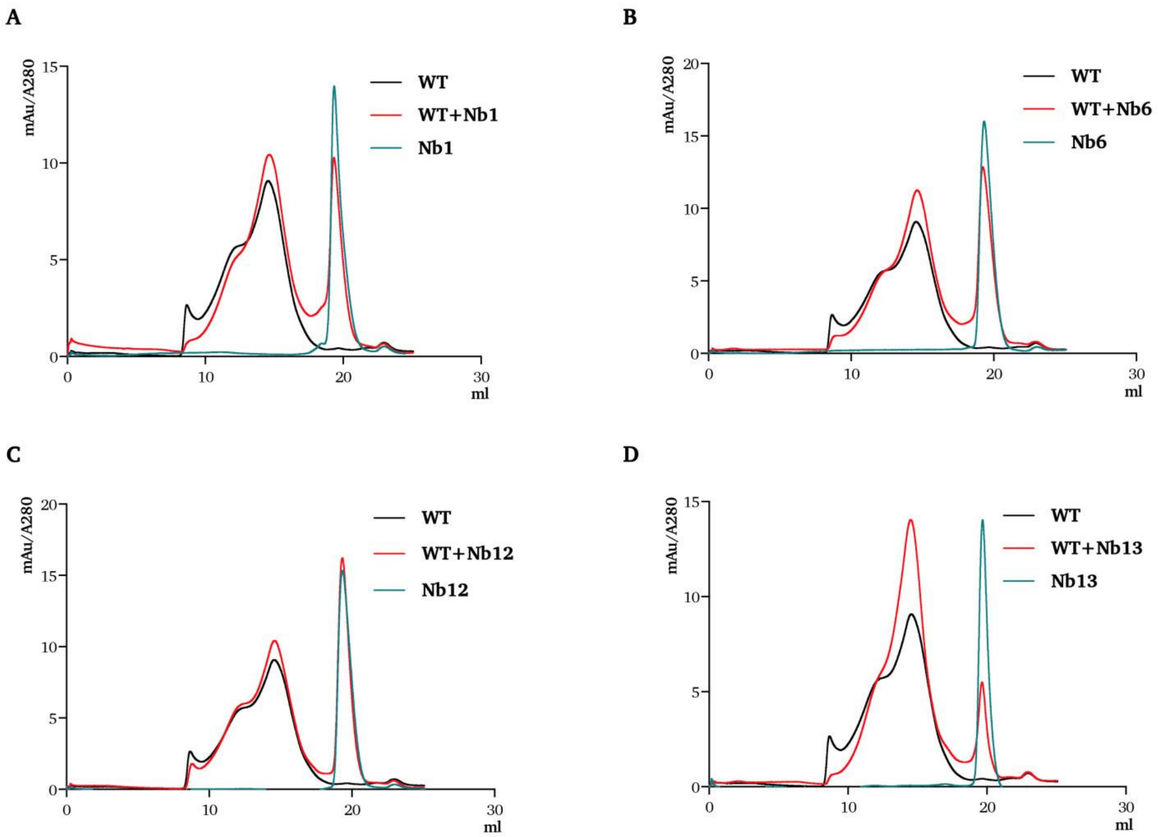

Supplementary Figure 11 │ Initial nanobody screening using size-exclusion chromatography. A-D. Representative elution profiles for the binding analysis of the selected nanobodies against LpCopA in the absence of Cu^{+}. The profile for the nanobody is shown in blue, LpCopA in black and the mixture in red. Due to the large size difference of LpCopA and the nanobodies, no peak shifts were detected. However, the absorbance intensity for the LpCopA-Nb13 complex increased compared to the non-complexed LpCopA-Nb12 elution profile, and correspondingly, the peak of the Nb13 nanobody reduced in the mixture. The absorbance intensity for the LpCopA-Nb1 and LpCopA-Nb6 complexes only displayed a minor increase compared to LpCopA only, indicating that Nb1 and Nb6 have low affinity for LpCopA in the absence of Cu^{+}.

Supplementary Figure 12

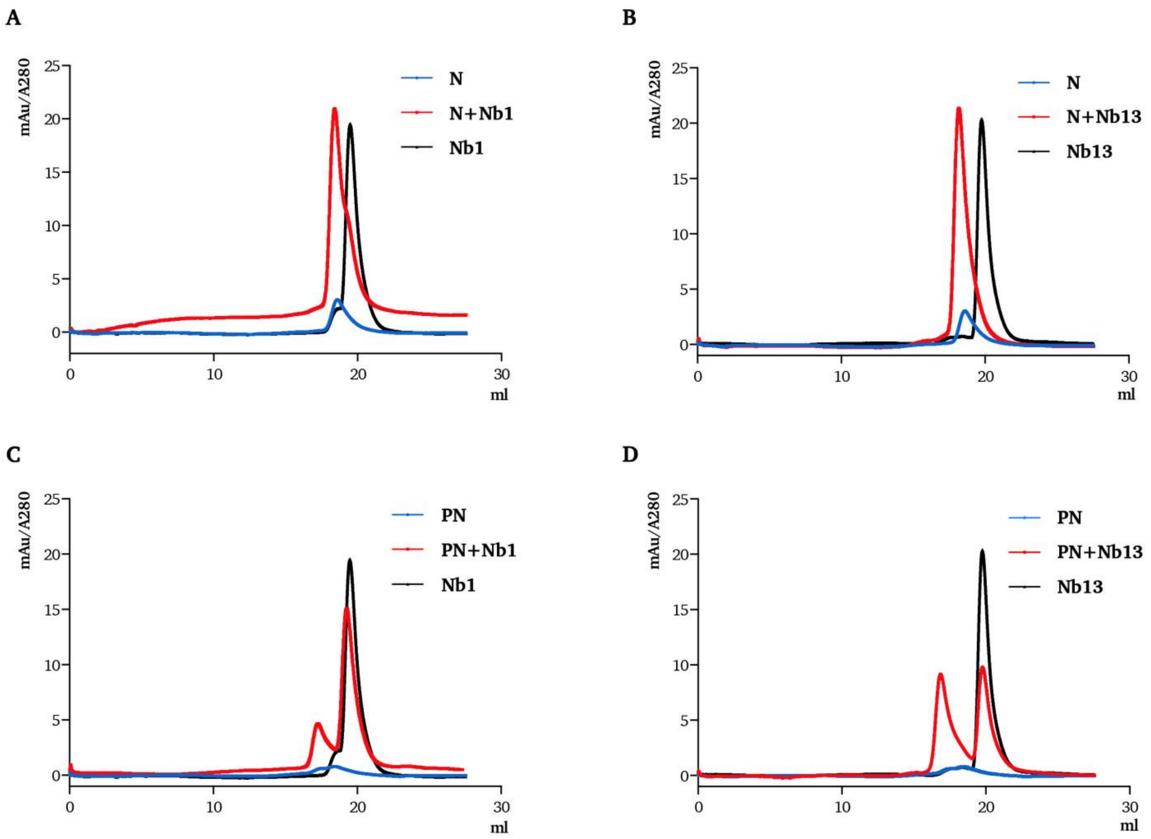

Supplementary Figure 12 │ Nanobody binding with three soluble domains of LpCopA. A-D. Representative elution profiles for the detailed binding analysis of Nb1 and Nb13 against soluble N- or PN-domains of LpCopA without Cu^{+}. The profile for the nanobody is shown in black, soluble domain only in blue, and the mixture in red. The molecular weight of the N-domain is ~13kDa and it forms as dimer in the profile, PN-domain is ~27kDa. The peak intensity of the complex increased significantly also shifted compared with Nbs only. The same amount of protein was loaded in C&D, whereas the peak of complex PN-Nb13 has increased more than PN-Nb1 complex, suggesting that Nb13 binding tightly with PN-domain.

Supplementary Figure 13

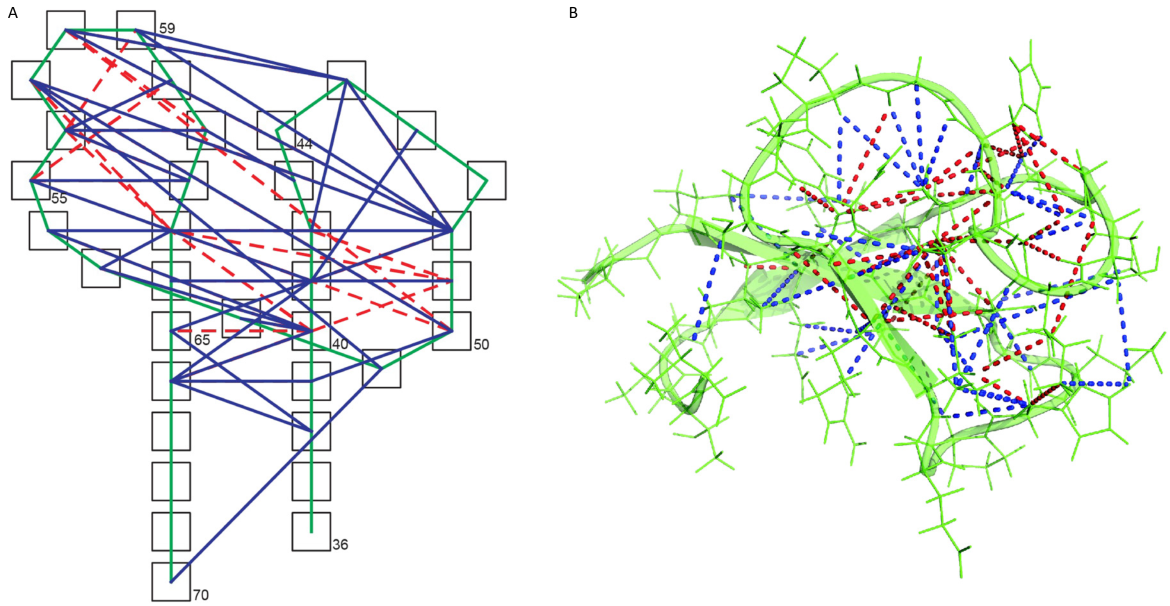

Supplementary Figure 13 │ Distribution of non-trivial distance constraints. A. Distance constraints used in the final iteration of the structure calculation for the apo form. Distances between protons with a separation in primary sequence of 3 residues or more are displayed in the topology diagram as blue full and red dashed lines for unique (71) and ambiguously assigned (63) NOE constraints, respectively. For the ambiguous distance constraints, only the distance corresponding to the assignment with the highest probability rank is shown. **B.** Distance constraints visualized on the structure of the apo-LpMBD.

Supplementary Table 1

Supplementary Table 1 | Primers used in this study.

Primer name	Primer sequence
**22b-T2-F**	GAAGGAGATATACATATGCACCACCACCACCACCACCACGAGAATCTTTATTTTCAGGGCATGGAGGGGCCTATTGTTTATAC
**22b-T1-F**	GAAGGAGATATACATATGCACCACCACCACCACCACCACGAGAATCTTTATTTTCAGGGCACGGTTTCGGAGGTTGTGAGCC
**22b-R**	GTGGTGGTGGTGGTGCTCGAGTCATCATAGTGTCACTCTCTTTAG
**22b-A-F**	GAAGGAGATATACATATGCACCACCACCACCACCACCACGAGAATCTTTATTTTCAGGGCCAAACGGGTAGTGCCATCCGTG
**22b-A-R**	GTGGTGGTGGTGGTGCTCGAGTCATCACTGAATCGGCGCACGGCTTCG
**22b-PN-F**	GAAGGAGATATACATATGCACCACCACCACCACCACCACGAGAATCTTTATTTTCAGGGCGTATTGATTAAAAATGCCG
**22b-PN-R**	GTGGTGGTGGTGGTGCTCGAGTCATCAACGCAAATCTCCATGCAAC
**22b-N-F**	GAAGGAGATATACATATGCACCACCACCACCACCACCACGAGAATCTTTATTTTCAGGGCGAAGGTCACCCTAAATTGACAC
**22b-N-R**	GTGGTGGTGGTGGTGCTCGAGTCATCAGTCTTCAACCACCAAGAG
**22b-Nbs-1F**	CGCTGCCCAGCCGGCGATGGCCATGCAGTTGCAGCTCGTGGAG
**22b-Nbs-2F**	CGCTGCCCAGCCGGCGATGGCCATGCAGGTGCAGCTCGTGGAG
**22b-Nbs-R**	GTGGTGGTGGTGGTGCTCGAGGCCCTGAAAATAAAGATTCTCTGAGGAGACGGTGACCTG
**SXXS-F**	GCCCCTGGTCATTCTCCTCTTTCTGGTATGGCTTTAGAAC
**SXXS-R**	GTTCTAAAGCCATACCAGAAAGAGGAGAATGACCAGGGGC
**AXXA-F**	CAATCTGCCCCTGGTCATGCTCCTCTTGCTGGTATGGCTTTAGAAC
**AXXA-R**	GTTCTAAAGCCATACCAGCAAGAGGAGCATGACCAGGGGCAGATTG
**MBD(T2-C212A)-F**	CGGTGACGGTTTAGGAGGTTGTGAGC
**MBD(T2-C212A) -R**	GCTCACAACCTCCTAAACCGTCACCG

Supplementary Table 2

Supplementary Table 2 | Restraint and structure statistics for the apo- and Ag^{+}-bound LpMBD NMR structures.

**Restraint statistics**	All (unambiguous)
Distance constraints [a]	
Total	472(301)/415(252)
Intra-residue	174(123)/160(112)
Sequential	145(95)/119(66)
Medium range	36(21)/26(17)
Long range	117(62)/110(57)
Dihedral angle	46/44
Hydrogen bonding	8/8
Distance violations	0.4
**Structure statistics**	
r.m.s.d.(Å) [b]	1.17/1.30
Angle rms (Å)[c]	0.889
Bond rms (°)[c]	0.0052
NOE target distance (Å)[c]	0.0566
Ramachandran statistics	
Most favourable regions	80.0/88.0 %
Additionally allowed	12.0/8.0 %
Generously allowed	4.0/0.0 %
Disallowed regions	4.0/4.0 %

[a] Long-range meaning that the residue difference, D, was 5 or more and medium range; $1<D<5$.

[b] Coordinate rmsd calculated for 10 ensemble members

[c] Statistics are averaged for the apo- and Ag^{+}-bound form of the protein
